# Mechanistic study of ATP-dependent regulation of the Hrq1 oligomeric state and its DNA unwinding activity in *Saccharomyces cerevisiae*

**DOI:** 10.1016/j.jbc.2026.113404

**Published:** 2026-08-07

**Authors:** Xiao Zhu, Yangxue Dai, Yan Xie, Hailei Guo, Zhe Wang, Hua Zhao, Zhisong Wang, Yi Zhang, Yang Liu, Bo Zhang

**Affiliations:** 1 College of Basic Medicine, Zunyi Medical University, Zunyi, Guizhou Province, China; 2 School of Public Health, Zunyi Medical University, Zunyi, Guizhou Province, China; 3 Zunyi Center for Disease Control and Prevention, Zunyi, Guizhou Province, China

**Keywords:** DNA unwinding mechanism, Hrq1 helicase, oligomerization, RecQ helicase, ATP-dependent regulation

## Abstract

The yeast Hrq1 protein is an important model for studying the human RECQL4 helicase and RECQL4-associated diseases. In this study, we simultaneously isolated and characterized two stable oligomeric states of Hrq1 expressed in insect cells: a monomeric Hrq1 and a higher-order oligomeric fraction. Intriguingly, ATP not only promotes the dissociation of the oligomeric fraction into monomeric or smaller Hrq1-containing species but also markedly stimulates the DNA-unwinding activities of both forms. Furthermore, Hrq1-mediated unwinding is strictly dependent on a forked DNA substrate, and its activity increases with increasing lengths of either the 5′- or 3′-single-stranded DNA tail. Based on the limited unwinding processivity of Hrq1, we propose a “relay-style” mechanism in which an initial Hrq1 monomer partially unwinds the duplex and subsequently dissociates, allowing additional Hrq1 monomers to bind the newly exposed 3′ single-stranded region and continue the unwinding reaction. However, this process can be inhibited by *Saccharomyces cerevisiae* replication protein A through competition for the exposed single-stranded DNA. These findings provide new insights into the oligomeric-state-dependent regulation and unwinding mechanism of Hrq1 and may contribute to a broader understanding of RecQ family helicases.

## Introduction

RecQ family helicase are highly conserved from bacteria to humans and play critical roles in maintaining genome stability (1, 2, 3, 4). In humans, mutations in the genes encoding RECQL1, BLM, WRN, and RECQL4 can lead to various genetic syndromes, many of which are characterized by progeroid features and/or increased cancer susceptibility (5, 6, 7, 8). In-depth investigation of these syndromes largely depends on suitable model systems. Yeast models offer notable advantages because their DNA repair pathways are highly conserved (9). However, yeasts were traditionally thought to encode only a single RecQ family helicase—Sgs1 in budding yeast or Rqh1 in fission yeast—both of which are orthologs of human BLM, while lacking clear orthologs of human RECQL1, WRN, and RECQL4. This apparent absence has hindered the development of yeast models for the corresponding human diseases (10, 11).

In a genome-wide screen, the yeast gene *YDR291W* was identified as an important factor involved in the DNA damage response, and its encoded protein shares significant sequence similarity with human RECQL4 (12). Subsequent bioinformatic analysis identified a new family of RecQ4 helicase homologs in fungi and plants, designated Hrq1 (13). Notably, the gene encoding this protein in *Saccharomyces cerevisiae* is *YDR291W*, the same gene identified in the earlier screen. Consequently, Hrq1 has emerged as an important molecular model for studying disorders associated with RECQL4 loss of function, including Rothmund-Thomson syndrome.

Research based on this model has uncovered multiple biological functions of Hrq1. In telomere homeostasis, Hrq1 is constitutively enriched at telomeres and can directly bind telomeric DNA (14, 15). Its absence leads to increased aberrant telomere addition at DNA double-strand break sites, thereby exacerbating genomic instability (14). Additionally, Hrq1 maintains telomere length homeostasis in coordination with the Pif1 helicase through dynamic module formation (16, 17, 18) or regulation by the Cdc13 protein (19). In DNA damage repair, Hrq1 is a key factor in DNA interstrand crosslink repair (14, 20, 21). Genetic evidence indicates that it operates in the same repair pathway as the nuclease Pso2, independently of the Fanconi anemia pathway (14, 22). Mechanistically, Hrq1 binds and activates Pso2 through its N-terminal domain, markedly enhancing the nuclease activity of Pso2 on crosslinked DNA in an ATP hydrolysis-dependent manner (20, 21). This mechanism is evolutionarily conserved: human RECQL4 can functionally substitute for Hrq1 in yeast to activate Pso2 (21, 23), and the plant homolog AtHrq1 also exhibits conserved DNA repair activity (24). Together, these findings highlight the conserved role of Hrq1/RECQL4 family helicases in maintaining genome stability.However, basic biochemical studies of Hrq1, particularly those addressing its duplex-unwinding mechanism, remain limited, and several key questions remain unresolved. For example, it is still unclear how 3′ single-stranded DNA ends regulate Hrq1 enzymatic activity (25, 26) and whether the oligomeric state of Hrq1 depends on the expression system used (14, 15).

This study aimed to characterize the oligomeric states and DNA-unwinding activity of Hrq1. In contrast to previous studies (14, 15), we simultaneously isolated two stable forms of Hrq1 expressed in insect cells: a monomeric Hrq1 (Hrq1mon) and a higher-order oligomeric fraction (Hrq1-oligo). We further found that ATP promoted the dissociation of Hrq1-oligo into monomeric or smaller Hrq1-containing species. Using an optimized stopped-flow fluorescence resonance energy transfer (FRET) assay, we demonstrated that Hrq1-mediated unwinding was strictly dependent on a forked DNA substrate containing a 5′single-stranded tail and that unwinding efficiency increased with increasing tail length. Based on the limited unwinding processivity of Hrq1, we propose a “relay-style” mechanism in which successive Hrq1 monomers continue the unwinding reaction. This process was efficiently inhibited by the single-stranded DNA-binding protein *Saccharomyces. cerevisiae* replication protein A (ScRPA) through competition for the exposed single-stranded DNA. Together, these findings define key biochemical properties of Hrq1 and provide new insights into its oligomeric-state-dependent regulation and DNA-unwinding mechanism.

## Results

### ATP Induces the Dissociation of higher-order Hrq1 assemblies into smaller species

**Figure 1:**
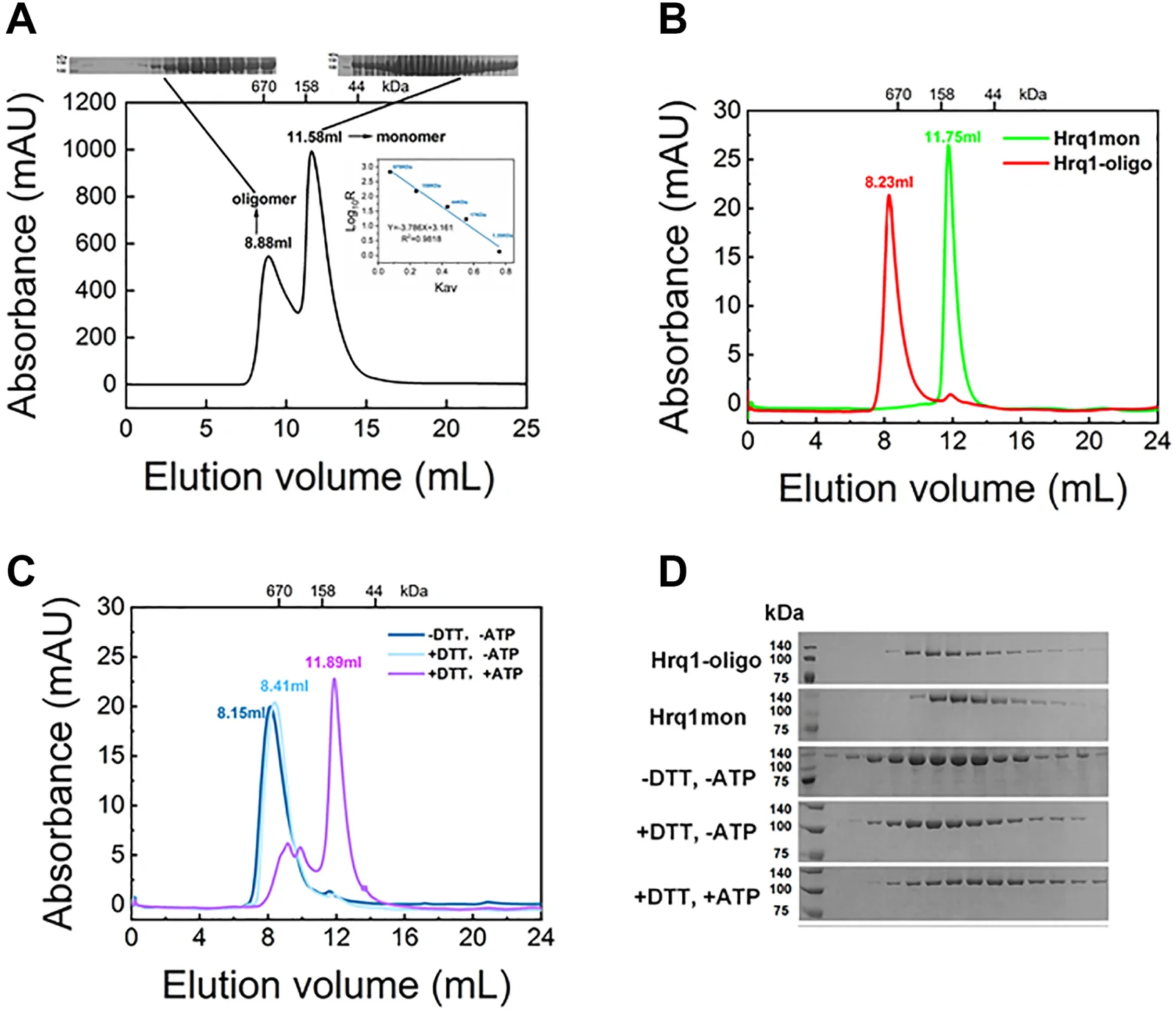
**ATP Induces the Dissociation of higher-order Hrq1 assemblies into monomer-like species.** Note: *A,* SEC profile of WT Hrq1 protein showing two continuous absorbance peaks at 280 nm. Inset: standard curve generated using molecular mass markers for SEC analysis. *B,* SEC reanalysis of the first elution peak (4.77 mg/ml) and the second elution peak (5.23 mg/ml). The first peak eluted earlier than the largest molecular mass standard, thyroglobulin (670 kDa), suggesting that this fraction represents a Hrq1 species of high molecular mass near or beyond the upper limit of the calibration range. The second elution peak corresponded to an apparent molecular weight of approximately 148 kDa, close to the theoretical molecular mass of monomeric Hrq1. *C,* SEC analysis of the redistribution of the higher-order Hrq1 fraction toward smaller species induced by ATP. Hrq1 protein at 1.14 mg/ml was incubated with 5 mM ATP at 25 °C in buffer containing 1 mM DTT. After ATP treatment, the absorbance peak at 280 nm shifted toward a larger elution volume, similar to that of the monomeric or monomer-like Hrq1 fraction. *D,* SDS-PAGE gel confirming that the elution peak fractions in *panels B* and *C* contained Hrq1 as the major protein species. SEC, size-exclusion chromatography.

Previous studies reported that Hrq1 forms a heptamer when expressed in *E. coli* but remains monomeric when expressed in insect cells. This difference has been attributed to the absence of eukaryotic post-translational modifications in prokaryotic expression systems, which may influence the assembly state of Hrq1 (14, 15). However, size-exclusion chromatography (SEC) of Hrq1 expressed and purified from insect cells revealed two distinct A280 peaks in the elution profile. SDS-PAGE analysis confirmed that fractions from both peaks contained Hrq1 (Fig. 1*A*), indicating that Hrq1 can exist in both monomeric or monomer-like species and higher-order assembly states in a eukaryotic expression system. To estimate the apparent molecular masses of these two Hrq1 forms in solution, we collected the fractions corresponding to each peak and subjected them to a second round of SEC analysis. Based on the molecular mass calibration curve (Fig. 1*A*, inset), the first peak appeared near or beyond the upper limit of the calibration range, defined by the largest standard, 670-kDa thyroglobulin, suggesting the presence of a high-molecular-mass Hrq1-containing species. It should be noted that the high-molecular-mass peak eluted near or beyond the upper limit of the SEC calibration range, which was defined by the largest protein standard used, 670-kDa thyroglobulin. Therefore, its apparent molecular mass is associated with substantial uncertainty, and these data cannot be used to determine the exact molecular mass or oligomeric stoichiometry of this species. Nevertheless, its early elution position indicates the presence of a higher-order Hrq1-containing assembly, consistent with the other characterization data (Fig. 1, *A* and *B*). The second peak had an apparent molecular mass of approximately 148 kDa, which was reasonably close to the theoretical molecular mass of a Hrq1 monomer, approximately 123 kDa (Fig. 1*B*).

Because ATP-induced dissociation of oligomeric assemblies has been reported for other RecQ family helicases (27, 28), and because the subsequent helicase assays were performed in a reducing buffer containing DTT, we examined the effects of ATP and DTT on the oligomeric state of Hrq1. Hrq1 was adjusted to a concentration of 1.14 mg/ml and initially analyzed by SEC in the absence of both DTT and ATP. Under these conditions, the major peak eluted at approximately 8.15 ml, corresponding to the high-molecular-mass Hrq1 fraction. Addition of 1 mM DTT caused only a slight shift in the peak position, from approximately 8.15 to 8.41 ml, and the peak remained within the high-molecular-mass elution range. Thus, DTT alone did not induce substantial dissociation of the high-molecular-mass Hrq1 fraction under the conditions tested. By contrast, following the addition of 5 mM ATP and incubation at 37 °C in the presence of 1 mM DTT, the major A280 peak shifted markedly to approximately 11.89 ml, close to the elution position of the monomeric or monomer-like Hrq1 fraction. A minor portion of the A280 signal remained within the earlier elution range of approximately 8.41 to 10.00 ml, indicating the persistence of residual high-molecular-mass Hrq1 species (Fig. 1*C*). SDS-PAGE analysis confirmed the presence of Hrq1 in all collected fractions (Fig. 1*D*). Together, these results indicate that ATP promotes the redistribution of the high-molecular-mass Hrq1 fraction toward monomer-like or other smaller Hrq1-containing species, although the transition is incomplete.

To further characterize the oligomeric properties of Hrq1, we used dynamic light scattering (DLS) to measure the hydrodynamic radii and estimate the apparent molecular masses of the isolated monomeric or monomer-like and higher-order Hrq1 fractions in the presence or absence of ATP and ssDNA. DLS analysis showed that, in the absence of ATP, the higher-order Hrq1 fraction remained as large assemblies regardless of whether 10- or 56-nt ssDNA (dT10 or dT56) was present, with hydrodynamic radius (Rh) of approximately 10 to 11 nm and estimated apparent molecular masses greater than 700 kDa (Table 1 and Fig. S2). After the addition of ATP, both the Rh and apparent molecular mass decreased markedly, supporting the conclusion that ATP shifts higher-order Hrq1 assemblies toward smaller species, including monomer-like species or monomer-enriched populations (Table 1). Notably, when both ATP and ssDNA were present, the apparent molecular mass of the resulting Hrq1-containing species depended on ssDNA length: after incubation with ATP and dT_10_, the apparent molecular mass of Hrq1 decreased from approximately 754 kDa to approximately 167 kDa, whereas in the presence of ATP and dT56, it was approximately 517 kDa. We therefore hypothesize that ATP first promotes the dissociation of higher-order Hrq1 assemblies into smaller species, which subsequently associate with ssDNA to form Hrq1-containing nucleoprotein complexes of different apparent sizes, depending on the length or scaffolding capacity of the ssDNA (Table 1). To examine this possibility, we analyzed the interaction between the pre-isolated monomeric or monomer-like Hrq1 fraction and ssDNA. The results showed that the molecular mass of this fraction did not change substantially following incubation with dT10 (approximately 114 kDa, compared with the theoretical molecular mass of 123 kDa for an Hrq1 monomer), whereas incubation with dT56 increased the apparent molecular mass to approximately 437 kDa. This value was close to that observed under the “higher-order Hrq1 + ATP + dT_56_” condition, supporting the possibility that multiple Hrq1 monomers or smaller Hrq1 species can bind along longer ssDNA to form larger nucleoprotein complexes (Table 1).

**Table 1:** Parameters for the Hrq1 helicase in the absence or presence of ATP and DNA obtained from dynamic light scattering measurements

Substrate/ligands	Concentration	Radius (nm)	Apparent hydrodynamic molecular Mass
Hrq1-oligo^a^	1 μM	10.1 ± 0.4	753.93 ± 5.16
Hrq1-oligo + ATP	1 μM + 5 mM	6.3 ± 0.2	249.85 ± 6.22
Hrq1-oligo + dT_10_	1 μM + 3 μM	9.8 ± 0.5	702.57 ± 8.69
Hrq1-oligo + dT_56_	1 μM + 3 μM	11.2 ± 0.4	960.27 ± 7.65
Hrq1-oligo + ATP + dT_10_	1 μM + 5 mM + 3 μM	5.3 ± 0.3	166.73 ± 2.63
Hrq1-oligo + ATP + dT_56_	1 μM + 5 mM + 3 μM	8.6 ± 0.1	517.28 ± 3.37
Hrq1mon^b^	1 μM	4.7 ± 0.2	125.87 ± 1.46
Hrq1mon + dT_10_	1 μM + 3 μM	4.5 ± 0.1	113.69 ± 5.16
Hrq1mon + dT_56_	1 μM + 3 μM	8.0 ± 0.6	436.97 ± 10.11

a Hrq1-oligo represents the oligomeric/high-molecular-weight fraction of Hrq1, without assignment to a specific oligomeric state.

b The theoretical molecular weight of the Hrq1mon is 123 kDa. Hrq1-oligo, higher-order oligomeric fraction; Hrq1mon, monomeric Hrq1.

Together, the SEC and DLS results suggest that, in the eukaryotic expression system, Hrq1 exists in monomeric or monomer-like states as well as in higher-order assembly states, and that ATP shifts higher-order Hrq1 assemblies toward monomer-like species or other smaller assemblies. The resulting smaller species can then associate with ssDNA of different lengths to form Hrq1-containing nucleoprotein complexes of varying apparent sizes. However, because SEC and DLS report apparent hydrodynamic sizes rather than precise stoichiometry, these data cannot unambiguously distinguish between a complex containing a pre-existing Hrq1 oligomer and a nucleoprotein complex formed by multiple Hrq1 monomers or smaller species associating with a longer ssDNA molecule. Therefore, we interpret the high-molecular-mass Hrq1–ssDNA species cautiously as Hrq1-containing nucleoprotein complexes without assigning them a defined oligomeric stoichiometry.

### General biochemical properties of Hrq1

**Figure 2:**
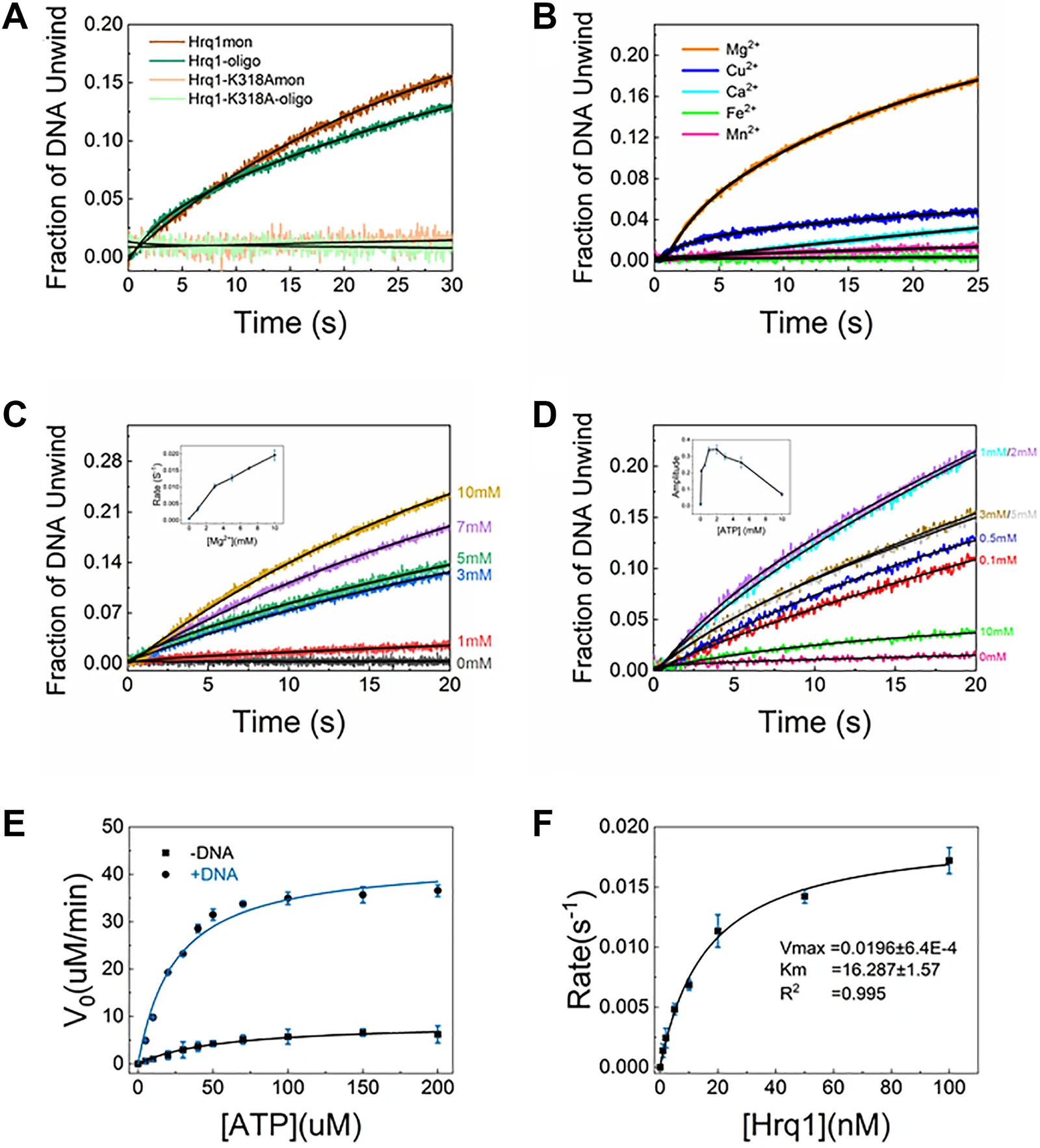
**General characterization of Hrq1 helicase activity.** Note: All DNA unwinding reactions shown in panels *A and D* were performed under multiple-turnover conditions, because no protein trap was included unless otherwise indicated. The stopped-flow traces in panels *A and D* were fitted using a biexponential equation, as described in section 4.10, “Stopped-Flow FRET Measurements,” of Materials and Methods. *A,* activity verification of Hrq1 and its catalytically inactive mutant. After pre-incubation at 37 °C for 5 min, 1 mM ATP was added to initiate the reaction. *B,* effect of divalent cations on Hrq1 helicase catalysis. Reaction conditions: 50 mM NaCl, 25 mM Tris-HCl (pH 7.5), 1 mM ATP; the influence of 5 mM metal ions (without DTT) on Hrq1 helicase catalysis was tested. *C,* concentration-dependent effect of Mg^2+^ on the apparent Hrq1 unwinding rate. The reaction buffer contained 50 mM NaCl,1 mM DTT, and 25 mM Tris-HCl (pH 7.5), and the Mg^2+^ concentration was varied as indicated. Inset: Relationship between the Mg^2+^ concentration and the apparent unwinding rate. *D,* effect of ATP concentration on Hrq1 helicase activity.The ATP concentration was varied under the indicated reaction conditions. Inset: Relationship between the ATP concentration and the unwinding amplitude. *E,* ATPase activity of Hrq1. The initial rates of ATP hydrolysis at different ATP concentrations were measured using 100 nM Hrq1 in the presence or absence of 1 μM forked DNA substrate (D20-3′dT25-Fk). Reactions were carried out at 25 °C in a buffer containing 50 mM NaCl, 1 mM DTT, 25 mM Tris-HCl (pH 7.5), and 10 mM MgCl_2_. The experimental data were fitted to the Michaelis–Menten equation. *F,* Michaelis–Menten analysis of the DNA-concentration dependence of Hrq1-mediated unwinding. In the presence of 2 mM ATP, with the DNA concentration varied as indicated, Hrq1 exhibited a *V*_max_ of 0.0196 ± 6.4 × 10^−4^ s^−1^ and an apparent *K*_m_ for DNA of 16.29 ± 1.57 nM.

After obtaining highly purified Hrq1 protein (Fig. S3*A*) and characterizing its oligomeric properties, we first assessed its helicase activity using a stopped-flow FRET assay (Fig. 2*A*). The results showed that both the monomeric or monomer-like and the higher-order fractions of WT Hrq1 exhibited readily detectable helicase activity, whereas the corresponding K318A variants showed no detectable helicase activity. This result not only confirmed the essential role of the conserved Lys-318 residue within the Walker A motif in the catalytic activity of Hrq1 (14, 17, 29), but also argued against nonspecific disruption of the DNA substrate as the source of the observed unwinding signal.

To optimize the DNA-unwinding conditions, we used monomeric Hrq1 and a forked DNA substrate containing a 20-bp duplex and 25-nt 3′ and 5′ poly(dT) tails (D20-3′dT25-Fk; Table S1). We systematically evaluated the effects of temperature, pH, DTT concentration, and other reaction parameters on Hrq1 catalytic activity. Using the optimized conditions (37 °C, pH 7.5, 1 mM DTT; Fig. S3, *B*–*D*), we further screened different divalent metal ions and examined the concentration-dependent effects of Mg^2+^ and ATP. Among the divalent metal ions tested, only Mg^2+^ supported robust Hrq1-mediated unwinding, whereas Ca^2+^, Cu^2+^, Mn^2+^ and Fe^2+^ failed to support comparable unwinding activity (Fig. 2*B*). Zn^2+^ and Ni^2+^ caused a notable decrease in the fluorescence signal (Fig. S3*E*). However, this decrease could not be attributed directly to inhibition of Hrq1 helicase activity and likely reflected metal-ion-dependent perturbation of the fluorescence readout, potentially through effects on the fluorophores and/or DNA substrate. Having identified Mg^2+^ as the only tested divalent metal ion that supported robust unwinding, we investigated its concentration-dependent effect over a range of 0 to 10 mM. Hrq1 activity increased with increasing Mg^2+^ concentration and approached saturation at approximately 10 mM (Fig. 2*C*). This concentration was of the same order of magnitude as the reported intracellular Mg^2+^ concentration range in the *S. cerevisiae* cytosol (15–30 mM) (30, 31). Notably, when ScPif1 was used as a control (Fig. S3*F*), its activity decreased markedly at high Mg^2+^ concentrations, whereas Hrq1 retained substantial activity, indicating greater tolerance to elevated Mg^2+^ concentrations. Analysis of the ATP concentration dependence revealed a clear bell-shaped response in Hrq1 activity (Fig. 2*D*). The activity peaked at 1 to 2 mM ATP with unwinding amplitude of 34.1%, but decreased sharply at ATP concentrations above 2 mM, with a maximum reduction of 79.4% relative to the peak activity. This bell-shaped ATP dependence was reproducibly observed across independent kinetic measurements, with eight kinetic traces collected at each ATP concentration. The ATP concentration associated with maximal Hrq1 activity was close to the reported intracellular ATP concentration of approximately 2 mM (32), although this correspondence alone does not establish physiological adaptation. However, the decrease in Hrq1 activity at high ATP concentrations should not be interpreted simply as classical substrate inhibition or direct ATP-mediated inhibition. For most DNA helicases, the catalytically relevant substrate is the Mg^2+^–ATP complex rather than free ATP. Therefore, when the total Mg^2+^ concentration is held constant while the ATP concentration is increased, the equilibrium among free Mg^2+^, Mg^2+^–ATP, and free ATP may be altered. Previous biochemical characterization of *E. coli* RecQ helicase also showed that DNA helicase activity can be influenced by ATP concentration, free Mg^2+^ concentration, and other reaction components (33). Thus, the reduced Hrq1 activity at high ATP concentrations may partially reflect changes in Mg^2+^–ATP availability, free Mg^2+^ concentration, or the Mg^2+^/ATP ratio rather than a direct effect of ATP alone. In contrast, ScPif1 activity approached saturation at high ATP concentrations without showing a comparable decline (Fig. S3*G*), indicating that ScPif1 and Hrq1 respond differently to changes in ATP concentration. Together, these results suggest that Hrq1 is particularly sensitive to the ATP/Mg^2+^ cofactor environment, and that its bell-shaped ATP dependence may reflect the combined effects of Mg^2+^–ATP availability, free Mg^2+^ concentration, and the Mg^2+^/ATP balance.

To characterize the catalytic mechanism of Hrq1, we systematically determined its ATP hydrolysis kinetics in the absence and presence of DNA. It should be noted that the *K*_m_,app values for ATP reported here should not be interpreted directly as equilibrium dissociation constants for ATP binding (*K*_ᴅ_). Nevertheless, according to classical enzyme kinetics, under rapid-equilibrium conditions, or when the catalytic step is slow relative to substrate dissociation, that is, when *k*_cat_ < *k*_off_, *K*_m_,app can approximate the equilibrium dissociation constant (*K*_ᴅ_) of the enzyme–substrate complex (34, 35, 36).In the absence of DNA, Hrq1 exhibited relatively low basal ATPase activity (the *K*_m_,app for ATP = 56.9 ± 7.9 μM, *k*_cat_ = 86.5 ± 3.7 min^−1^; Fig. 2*E*). However, in the presence of a DNA substrate, Hrq1 ATPase activity was markedly stimulated: the *K*_m_,app for ATP decreased to 24.6 ± 3.5 μM, while *k*_cat_ increased to 432 ± 5.9 min^−1^. Thus, DNA increased the ATP hydrolysis rate by approximately fivefold and increased the apparent catalytic efficiency (k_cat_/K_m_,app) approximately 11-fold. Although we did not directly measure ATP-binding *K*_D_, the decrease in *K*_m_,app for ATP indicates more efficient ATP utilization by DNA-bound Hrq1 and, under rapid-equilibrium conditions (k_cat_ < k_off_, ATP), could be consistent with an increase in apparent ATP affinity. These results indicate that Hrq1 possesses strongly DNA-stimulated ATPase activity and that DNA binding promotes a catalytically more active ATPase state.

Notably, despite the enzyme concentration being much higher than the substrate concentration ([E]:[S] = 50:1) in the stopped-flow helicase assay, the maximum unwinding amplitude was only 34.1%. This limited yield, when considered together with the measured ATPase *k*_cat_ in the presence of DNA (432 min^−1^, approximately 7.2 s^−1^), suggests that ATP hydrolysis itself is not the only limiting factor for Hrq1-mediated unwinding. Rather, the low unwinding fraction likely reflects the relatively low processivity of Hrq1, premature dissociation from the DNA substrate, and/or nonproductive binding events (37, 38, 39, 40, 41). In other words, although DNA strongly stimulates Hrq1 ATPase activity, not every ATP hydrolysis event or enzyme-binding event is necessarily converted into productive duplex unwinding. This interpretation is also consistent with our observation that Hrq1 unwinding efficiency was markedly enhanced under multiple-turnover conditions (Fig. S4*B*), indicating that efficient unwinding requires repeated cycles of binding, dissociation, and rebinding.

Further steady-state unwinding analysis showed that, under conditions of 2 nM DNA substrate and 2 mM ATP, Hrq1 exhibited a *V*_max_ of 0.0196 ± 6.4 × 10^−4^ s^−1^ and an apparent *K*_m_ for the DNA substrate of 16.287 ± 1.57 nM (Figs. 2*F* and S3*H*). Collectively, these data reveal the coupling between ATPase activity and DNA unwinding by Hrq1, while also emphasizing that ATPase *k*_cat_ should not be directly equated with the single-molecule DNA translocation rate or base-pair unwinding rate.

### Hrq1 helicase activity depends on a forked DNA structure

As a member of the RecQ helicase family, Hrq1 unwinds DNA with 3′→5′ polarity (2, 8, 22, 25). Therefore, we used substrates with a fixed 25-nt 3′-ssDNA tail, which meets the reported 25-nt ssDNA-binding-site requirement of Hrq1 (15), and systematically varied the length of the 5′-ssDNA tail (0, 1, 5, or 25 nt). A blunt-ended double-stranded DNA substrate was also included to examine Hrq1’s dependence on a forked DNA structure.

**Figure 3:**
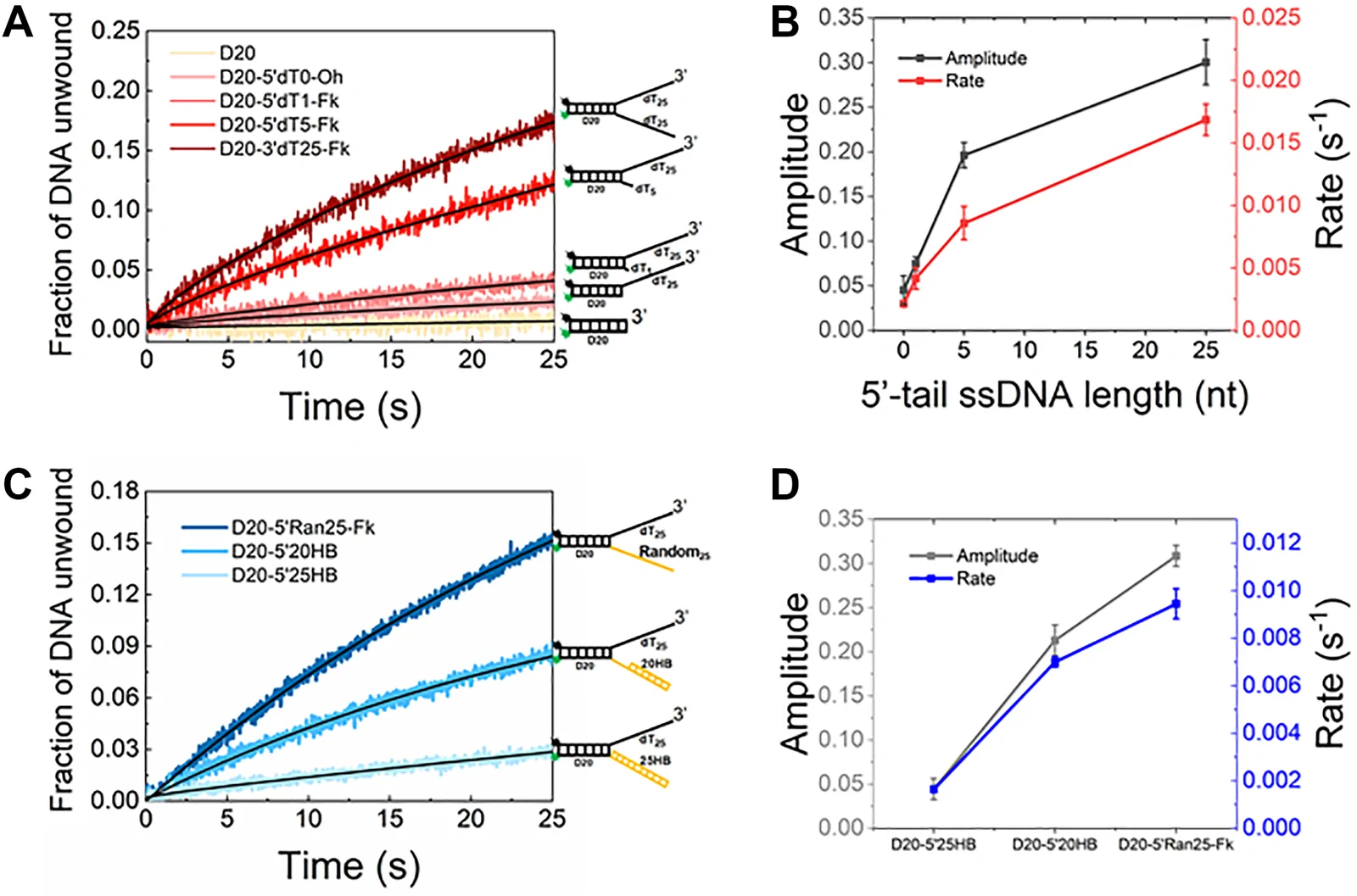
**Hrq1 exhibits fork-dependent helicase activity under multiple-turnover conditions.** Note: *A,* unwinding kinetics as a function of 5′-ssDNA tail length. Stopped-flow FRET was used to monitor the unwinding process of 20 bp forked DNA substrates (2 nM) containing a fixed 25-nt 3′-ssDNA tail and 5′-ssDNA tails of different lengths(0, 1, 5, or 25 nt). Hrq1 was used at a final concentration of 100 nM. The reaction buffer contained 25 mM Tris-HCl (pH 7.5), 50 mM NaCl, 10 mM MgCl_2_, and 1 mM DTT. Hrq1 and the DNA substrate were preincubated at 37 °C for 5 min in a two-syringe stopped-flow configuration, and the reaction was initiated by rapid mixing with 2 mM ATP. *Solid lines* represent biexponential fits to the data. The inset shows the substrate design strategy; D20 represents a blunt-ended double-stranded DNA substrate.*B,* relationship between 5′-ssDNA-tail length and the unwinding amplitude and apparent unwinding rate. Both parameters increased with increasing 5′-ssDNA-tail length. *C,* unwinding kinetics with a 5′-ssDNA tail changed from poly (dT_25_) to a random sequence, followed by full or partial sequence complementarity. The experimental conditions are the same as in panel *A*. *Solid lines* represent double-exponential function fit curves; *D,* the impact of fork structure integrity on unwinding amplitude and rate. All unwinding reactions shown in Figure 3 were performed under multiple-turnover conditions without the dT_56_ protein trap. Under these conditions, free or dissociated Hrq1 molecules were allowed to rebind the DNA substrate and participate in subsequent rounds of unwinding.

We first evaluated Hrq1’s helicase activity under single-turnover kinetic conditions. In this setup, excess Hrq1 (100 nM) was preincubated with 2 nM DNA substrate, and a high concentration of the protein trap oligonucleotide (1 μM dT_56_) was added to capture free Hrq1. The results showed that even in the presence of a fully forked DNA substrate, Hrq1 unwound only about 2.5% of the DNA (Fig. S4*A*). This very low unwinding efficiency suggests that a single round of substrate binding supports only limited duplex unwinding, consistent with the low processivity previously reported for Hrq1 (15). In sharp contrast, the unwinding efficiency increased significantly under multiple-turnover kinetic conditions. In the absence of the protein trap (dT_56_), the unwinding amplitude of the same substrate (D20-3′dT25-Fk) surged to 30.3 ± 2.53% (Fig. S4*B*). This result clearly indicates that efficient unwinding by Hrq1 depends on the continuous supply of free enzyme molecules in the reaction system. A single enzyme molecule cannot unwind the entire DNA substrate but requires the coordinated action of multiple free enzyme molecules to complete the unwinding process.Using the optimized multiple-turnover kinetic system, we systematically analyzed the effect of the 5′-ssDNA tail length on Hrq1 activity. As an important control, blunt-ended duplex DNA lacking an ssDNA tail showed almost no detectable unwinding activity, indicating that Hrq1 cannot efficiently initiate unwinding from a substrate lacking a single-stranded overhang (Fig. 3*A*). When the 5′ tail was absent or very short, Hrq1-mediated unwinding was also markedly reduced, whereas substrates containing both 3′- and 5′-ssDNA tails supported more efficient unwinding. As the length of the 5′-ssDNA tail increased from 1 nt to 25 nt, the unwinding amplitude increased from 7.46 ± 0.79% at 1 nt to 30.3 ± 2.53% at 25 nt, corresponding to an approximately 306% increase (Fig. 3*B*, Table 2), and the overall unwinding rate increased by more than sixfold (Fig. 3*B*, Table 2). These data indicate that Hrq1 strongly prefers forked DNA substrates and that the presence of a 5′-ssDNA tail substantially enhances unwinding, potentially by facilitating enzyme loading, substrate binding, and/or processed unwinding.

**Table 2:** Unwinding parameters of different substrates in the fork structure-dependent assay

S/P	D20-5′dT0-Oh	D20-5′dT1-Fk	D20-5′dT5-Fk	D20-3′dT25-Fk	D20-5′Ran25-Fk	D20-5′20HB	D20-5′25HB
Amplitude	0.0451 ± 0.016	0.0746 ± 0.0079	0.1962 ± 0.0142	0.3003 ± 0.0253	0.2914 ± 0.0119	0.2129 ± 0.0175	0.0444 ± 0.0121
Rate (s-1)	0.0021 ± 2.3 × 10^−4^	0.0041 ± 8.5 × 10^−4^	0.0086 ± 1.3 × 10^−3^	0.0169 ± 0.0013	0.094 ± 6.3 × 10^−4^	0.0070 ± 2.1 × 10^−4^	0.0016 ± 1.2 × 10^−4^

S, Substrate; P, Parameters.

To relate Hrq1’s ATPase activity to the DNA-unwinding kinetics shown in Figure 3, we measured its steady-state *k*_cat_ in the presence of the forked DNA substrate (D20-3′dT25-Fk). Hrq1 exhibited a *k*_cat_ of 432 ± 5.9 min^−1^ (approximately 7.2 ± 0.37 s^−1^), whereas in the absence of DNA, *k*_cat_ was 86.5 ± 3.7 min^−1^ (Fig. 2*E*). Although this *k*_cat_ represents the maximal steady-state turnover number for ATP hydrolysis under the conditions tested, the apparent unwinding rate constant measured for the same substrate under multiple-turnover conditions (approximately 0.017 ± 0.0013 s^−1^, Table 2) was substantially lower. likely reflects the contribution of additional steps to the overall unwinding reaction, including enzyme binding, ATP-dependent conformational changes, strand separation, and enzyme dissociation, indicating that productive duplex unwinding is not determined by ATP hydrolysis alone. To test whether repeated cycles of enzyme contribute to efficient unwinding, we performed single-turnover experiments by preincubating 100 nM Hrq1 with 2 nM DNA and sequestering free or dissociated with 1 μM dT_56_ as a protein trap. Under these conditions, all substrates exhibited very low unwinding amplitudes, with the highest value, observed for D20-3′dT25-Fk being approximately 2.5 ± 0.44% (Fig. S4*A*), compared with approximately 30.3 ± 2.53% under multiple-turnover conditions (Fig. S4*B*). These results indicate that multiple free Hrq1 molecules acting sequentially are necessary to unwind the duplex efficiently, directly supporting the requirement for multiple-turnover events to achieve productive unwinding.

To further substantiate this dependence on a forked DNA structure, we replaced the 25-nt poly(dT) 5′ tail with a random sequence to obtain the substrate D20-5′Ran25-Fk (Fig. 3*C*). Unwinding efficiency for this substrate was not significantly different from that for D20-3′dT25-Fk (unwinding amplitudes: 29.14 ± 1.19% and 30.03 ± 2.53%, respectively; Fig. S4*C*), indicating that the observed effect was primarily dependent on tail structure rather than nucleotide sequence. We next tested substrates in which the 5′tail was partially or fully base-paired to form a hairpin structure (D20-5′20HB and D20-5′25HB; Table S1). When the 5′ tail was fully base-paired (D20-5′25HB), both the unwinding amplitude (4.44 ± 1.21%) and the apparent unwinding rate constant (0.0016 ± 1.2 × 10^−4^ s^−1^) decreased to levels comparable to those observed for the substrate lacking a 5′ tail (D20-5′dT0-Oh; apparent rate constant, 0.0021 ± 2.3 × 10^−4^ s^−1^, unwinding amplitude, 4.51 ± 1.6%) (Fig. 3*D*, Table 2). By contrast, partial base-pairing (D20-5′20HB) resulted in significantly higher amplitude (21.29 ± 1.75%) and apparent rate constant (0.007 ± 2.1 × 10^−4^ s^−1^) than those of the fully paired substrate (∗p∗ < 0.001) (Fig. 3*D* and Table 2).The values obtained for D20-5′20HB were comparable to those obtained for the standard forked substrate containing a 5-nt 5′ tail (D20-5′dT5-Fk; unwinding amplitude, 19.62 ± 1.42%, apparent rate constant, 0.0086 ± 1.3 × 10^−3^ s^−1^) (∗p∗ < 0.01). These results further support the conclusion that Hrq1-mediated unwinding depends strongly on the presence of an accessible forked DNA structure.

**Figure 4:**
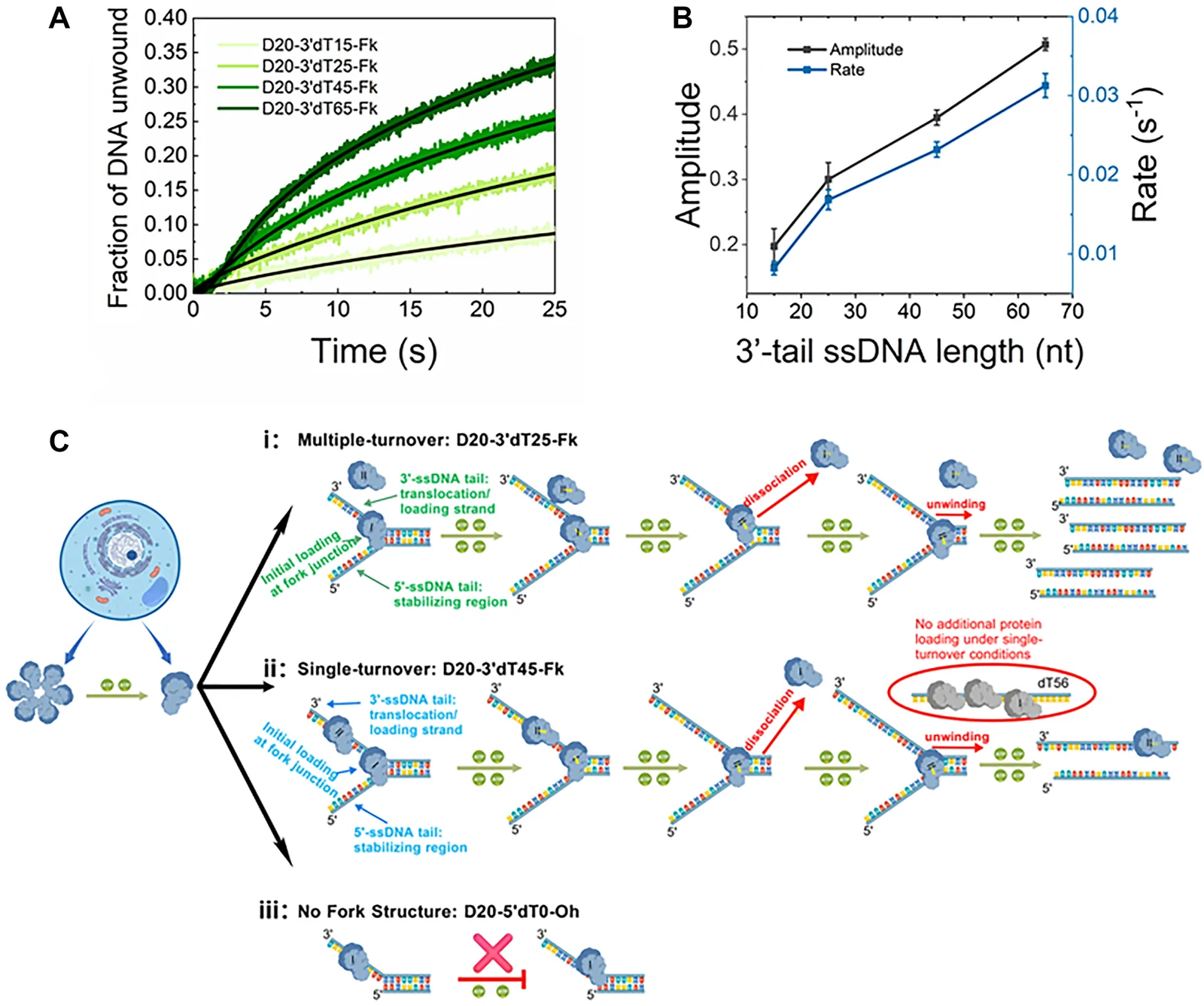
**The 3′-ssDNA tail stimulates Hrq1 helicase activity.** Note: *A,* real-time unwinding kinetics as a function of 3′-ssDNA tail length. Hrq1 (100 nM) unwound 20-bp forked DNA substrates (2 nM) containing 3′-ssDNA tails of different lengths (15, 25, 45, or 65 nt). The reaction conditions were the same as those described for Figure 3A. The reactions shown in panel A were performed under multiple-turnover conditions. *Solid lines* represent biexponential fits to the data, as described in Section 4.10, “Stopped-Flow FRET Measurements” of Materials and Methods. *B,* positive correlation between 3′-ssDNA tail length, unwinding amplitude, and apparent overall unwinding rate; *C,* proposed multiple-protein relay unwinding model of Hrq1. Mechanistic diagram illustrating Hrq1-mediated DNA unwinding under multiple-turnover, single-turnover, and no-fork conditions. (i) Under multiple-turnover conditions (D20-3′dT25-Fk), Hrq1 is initially loaded at the fork junction *via* the 3′-ssDNA tail (translocation/loading strand), while the 5′-ssDNA tail serves as a stabilizing region that enhances productive engagement at the fork. Free or dissociated Hrq1 molecules can repeatedly rebind newly exposed fork junctions, supporting continuous and efficient unwinding through iterative binding cycles. (ii) Under single-turnover conditions (D20-3′dT45-Fk) in the presence of the dT56 protein trap, free or dissociated Hrq1 is sequestered and cannot rebind the DNA substrate; therefore, efficient unwinding depends on a sufficiently long 3′-ssDNA loading strand capable of accommodating multiple prebound Hrq1 molecules, enabling sequential relay-like action. (iii) In the absence of a fork structure (D20-5′dT0-Oh), Hrq1 shows negligible unwinding activity, indicating that both the fork junction and the ssDNA tails are essential for productive helicase function. Created with BioGDP (61).

Our results demonstrate that Hrq1 helicase activity depends strongly on forked DNA substrates containing ssDNA tails, whereas blunt-ended duplex DNA supports little or no detectable unwinding (Fig. 3*A*). Although the presence of a 3′-ssDNA tail provides a loading site for Hrq1, the 5′-ssDNA tail further enhances unwinding efficiency in a length-dependent manner. These findings highlight Hrq1’s strong preference for forked DNA structures. Mechanistically, the 5′-ssDNA tail may enhance productive substrate engagement and/or facilitate repeated binding of Hrq1 molecules during relay-like unwinding, thereby increasing the overall unwinding efficiency. Specifically, these results indicate that the forked DNA structure, particularly the 5′-ssDNA tail and the ssDNA/dsDNA junction, is critical for efficient initiation of Hrq1-mediated unwinding (Fig. 3*A*). Although Hrq1 translocates in the 3′→5′ direction and the 3′-ssDNA tail provides the primary strand for loading and movement, substrates lacking an appropriate 5′-ssDNA tail exhibit very limited unwinding activity even under multiple-turnover conditions (Fig. 3*B*), indicating that repeated Hrq1 binding to the 3′-ssDNA tail alone is insufficient to support efficient duplex unwinding. Mechanistically, the 5′-ssDNA tail may act as an auxiliary loading or stabilizing region that promotes productive Hrq1 association at the fork junction, thereby facilitating unwinding initiation and subsequent relay-like unwinding. This interpretation is incorporated into the model in Figure 4*C*, which also illustrates that, in the absence of the 5′-ssDNA tail, Hrq1 fails to efficiently initiate or complete duplex unwinding (Figs. 3*A* and 4*C*).

### Hrq1 helicase activity is dependent on the length of the 3′ tail

Previous studies have established that Hrq1 strongly prefers forked DNA structures. However, those experiments used substrates with a fixed 25-nt 3′-ssDNA tail, and therefore did not address the effect of 3′ tail length on Hrq1-mediated unwinding (15). To systematically investigate the regulatory effect of 3′-ssDNA tail length on helicase activity, we designed a series of forked DNA substrates with a fixed 25-nt 5′-ssDNA tail, a 20-bp duplex, and 3′-ssDNA tails of different lengths (15, 25, 45, or 65 nt). The reaction conditions were identical to those used for the experiments shown in Figure 3*A*.

All tested substrates were unwound by Hrq1 (Fig. 4*A*). Notably, the unwinding amplitude increased significantly with increasing 3′-ssDNA tail length, rising from 19.75 ± 2.68% for the 15-nt tail to 50.68 ± 0.91% for the 65-nt tail, corresponding to an increase of approximately 157% (Fig. 4*B*). The overall unwinding rate also increased approximately fourfold, from 0.00821 ± 8.5 × 10^−4^ s^−1^ for the 15-nt tail to 0.0313 ± 0.00151 s^−1^ for the 65-nt tail (Fig. 4*B*). Because no protein trap (dT_56_) was included under the multiple-turnover conditions, free Hrq1 could repeatedly bind to the 3′ tail exposed upon enzyme dissociation, driving multiple proteins to sequentially open the entire double stranded DNA (Fig. 4*C*). To determine the effect of 3′-ssDNA tail length when rebinding by free Hrq1 was prevented, we performed single-turnover kinetic experiments. The results (Fig. S5) showed that substrates containing 3′-ssDNA tails of 45 nt or longer exhibited significantly greater unwinding amplitudes than the substrate containing a 25-nt 3′-ssDNA tail. One possible explanation is that the longer tails can accommodate more than one Hrq1 monomer, although the binding stoichiometry was not directly determined.

Regarding the ongoing debate in the literature over whether 3′-ssDNA tail length stimulates Hrq1 activity (25, 26), our experiments demonstrate that longer 3′-ssDNA tails (≥45 nt) significantly enhance helicase activity. We hypothesize that a sufficiently long 3′ tail (≥45 nt) provides multiple potential binding sites for Hrq1 monomers, thereby facilitating a relay-style sequential-binding mechanism (Fig. 4*C*). In this model, the initially bound protein unwinds a local DNA region and then dissociates, allowing a subsequent Hrq1 molecule to continue the unwinding process. A key feature of this model is that monomeric Hrq1 appears to serve as the functional unit. This interpretation is supported by the single-turnover results: when free or dissociated Hrq1 was sequestered using excess dT56 as a protein trap, unwinding of the substrate containing a 25-nt 3′ tail was markedly reduced, consistent with limited unwinding during a single round of Hrq1 binding. In contrast, 3′-ssDNA tails of at least 45 nt retained greater unwinding activity than the substrate with a 25-nt 3′-ssDNA tail, potentially because the longer tails may have allowed more than one Hrq1 molecule to bind before initiation of the reaction (Fig. S5). These results support the possibility that the additional binding capacity provided by a longer 3′ tail facilitates relay-style unwinding, although the number of Hrq1 molecules bound to each substrate was not directly determined. Additionally, this model provides a potential mechanism for partially overcoming the limited processivity of Hrq1: after one Hrq1 monomer unwinds a limited DNA region and dissociates, another monomer may bind to the available 3′-ssDNA region and continue the reaction, thereby extending the overall distance of duplex unwinding through successive binding events.

### The Hrq1 monomer exhibits greater helicase activity than the high-molecular-mass Hrq1 fraction

**Figure 5:**
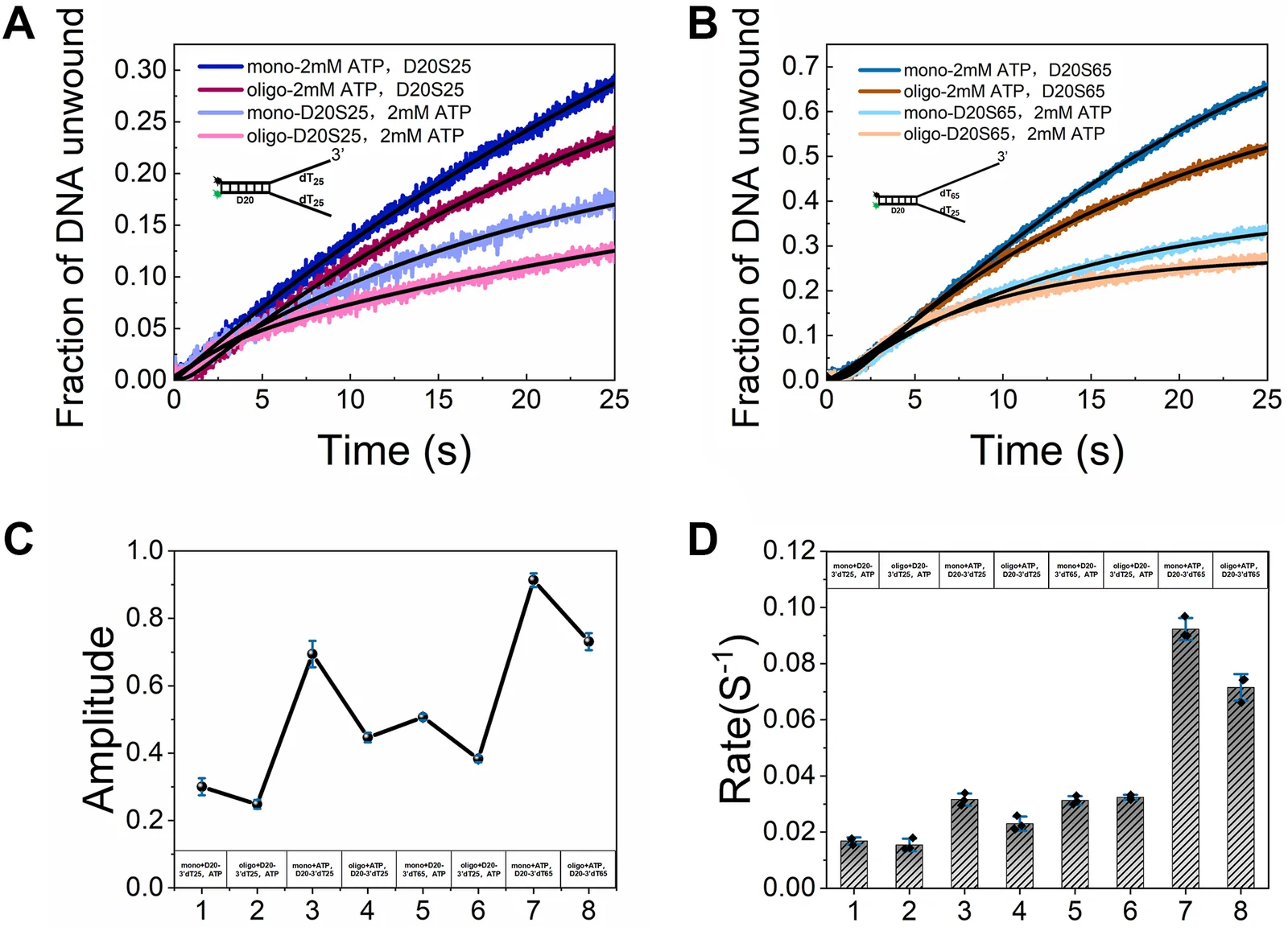
**Hrq1mon exhibits superior helicase activity compared with that of Hrq1-oligo.** Note: *A,* differential activation of Hrq1mon and the high-molecular-weight Hrq1 fraction (Hrq1-oligo) upon ATP preincubation. *A* stopped-flow FRET assay was used to monitor the unwinding process of the D20-3′dT25-Fk substrate (2 nM) by Hrq1mon and Hrq1-oligo (100 nM). Key variable: when Hrq1 was preincubated with 2 mM ATP in channel 1 for 5 min, instead of being preincubated with the substrate,its unwinding efficiency was significantly enhanced. *Solid lines* represent double-exponential fit curves, showing that ATP preincubation has a stronger activation effect on Hrq1mon than on Hrq1-oligo. Nonpreincubation controls: Hrq1-oligo + D20S25, 5 μM ATP; Hrq1-oligo + D20S25, 10 μM ATP. ATP-preincubation conditions: Hrq1-oligo + 5 μM ATP, D20S25; Hrq1-oligo + 10 μM ATP, D20S25. *B,* continued advantage of Hrq1mon activity on substrates with long 3′ tails. When the 3′-ssDNA tail was extended to 65 nt, Hrq1mon still exhibited higher unwinding efficiency than Hrq1-oligo, indicating that while long 3′ tails enhance the activity of both Hrq1-containing fractions, they do not alter the overall catalytic advantage of Hrq1mon. Nonpreincubation controls: Hrq1mon + D20S65, 5 μM ATP; Hrq1mon + D20S65, 10 μM ATP. ATP preincubation conditions: Hrq1mon +5 μM ATP, D20S65; Hrq1mon + 10 μM ATP, D20S65. *C and D,* hierarchical influence of incubation strategies on the unwinding amplitude and the dependence of apparent overall unwinding rate on Hrq1 assembly state and ATP preincubation mode. Hrq1mon, monomeric Hrq1; Hrq1-oligo, higher-order oligomeric fraction.

Given that ATP shifts the Hrq1-oligo toward monomeric or smaller Hrq1-containing species, previous activity studies have focused on the Hrq1 monomer. However, a direct comparison at equal protein concentrations (100 nM) revealed that the fraction (Hrq1mon) consistently exhibited greater unwinding activity (Fig. 5, *A* and *B*).

When we altered the preincubation order before reaction initiation, differential activation by ATP was observed. Preincubating Hrq1 with ATP (2 mM) at 37 °C for 5 min prior to adding the substrate resulted in significantly higher unwinding amplitudes and rates than those obtained with the standard protocol, in which the protein and substrate were preincubated before ATP addition. For the D20-3′dT25-Fk substrate, the monomer's unwinding amplitude increased from 30.03 ± 2.53% to 69.44 ± 3.91% (approximately 131.2% enhancement), and its rate increased from 0.0169 ± 0.0013 s^−1^ to 0.0316 ± 0.0021 s^−1^ (approximately 86.9% increase), showing a particularly pronounced activation. In contrast, the high-molecular-mass Hrq1 fraction showed a weaker response: its amplitude increased only from 24.81 ± 1.34% to 44.63 ± 1.41% (approximately 79.8%), and its rate from 0.0154 ± 0.0022 s^−1^ to 0.023 ± 0.0025 s^−1^ (approximately 49.3%) (Fig. 5, *C* and *D*, lanes one and three *versus* 2 and 4, Table 3).

**Table 3:** Comparison of Unwinding Parameters for Hrq1 Monomer and multimer under Various 3′-ssDNA Tail Lengths and Incubation Conditions

I/P	Hrq1Mon + D20-3′ dT25-Fk, ATP	Hrq1-oligo + D20-3′ dT25-Fk, ATP	Hrq1Mon + ATP, D20-3′dT25-Fk	Hrq1-oligo + ATP, D20-3′dT25-Fk	Hrq1Mon + D20-3′dT65-Fk, ATP	Hrq1-oligo + D20-3′ dT65-Fk, ATP	Hrq1Mon + ATP, D20-3′dT65-Fk	Hrq1-oligo + ATP, D20-3′dT65-Fk
Amplitude	0.3003 ± 0.0253	0.2481 ± 0.0134	0.6944 ± 0.0391	0.4463 ± 0.0141	0.5069 ± 0.0091	0.3829 ± 0.0097	0.9134 ± 0.0206	0.7311 ± 0.0254
Rate (s-1)	0.0169 ± 0.0013	0.0154 ± 0.0022	0.0316 ± 0.0021	0.0230 ± 0.0025	0.0313 ± 0.0015	0.03239 ± 8.6 × 10^−4^	0.0923 ± 0.0039	0.0715 ± 0.0047

I, Incubation Conditions; P, Parameters. Hrq1-oligo, higher-order oligomeric fraction.

To further determine whether this ATP-dependent activation is restricted to millimolar ATP concentrations, we also tested preincubation with much lower ATP concentrations. Notably, preincubation with either 5 μM or 10 μM ATP increased the unwinding amplitude by 58.05 ± 4.27% and 28.64 ± 1.91%, respectively (Fig. S7), indicating that ATP-mediated activation of Hrq1 can also occur at micromolar ATP concentrations. These results further support the idea that ATP preincubation promotes Hrq1 activation, likely through ATP-induced structural reorganization or conversion of the high-molecular-mass fraction into smaller, catalytically more competent species, rather than simply reflecting the catalytic effect of high ATP concentrations during the unwinding reaction. Notably, under ATP preincubation conditions, the unwinding rate of the monomeric Hrq1 fraction was significantly higher than that of the high-molecular-mass Hrq1 fraction. For the substrate with a 25-nt 3′-ssDNA tail, the difference in apparent unwinding rate was 0.0086 ± 8.31 × 10^−4^ s^−1^, corresponding to a 37.4 ± 3.44% higher rate for the monomeric fraction. Even with a 65-nt 3′-ssDNA tail, the monomer maintained an approximately 29% rate advantage (0.0923 ± 0.0039 s^−1^ *versus* 0.0715 ± 0.0047 s^−1^,Table 3). This consistent activity difference may be related to the assembly properties of the high-molecular-mass Hrq1 fraction: before engaging efficiently in DNA unwinding, Hrq1-oligo may require ATP-dependent conversion into monomeric or smaller catalytically competent species, resulting in a relative lag in the reaction.

These findings are consistent with the common regulatory mechanism in the RecQ family helicase, in which ATP binding and hydrolysis not only provide energy for catalysis but may also induce changes in the protein assembly state, such as redistribution of multimeric assemblies toward smaller, catalytically active species (27, 28). Combined with the earlier observation that ATP promotes the redistribution of Hrq1-oligo toward smaller Hrq1-containing species, these results suggest that the high-molecular-mass Hrq1 fraction is less readily activated than Hrq1mon under the tested conditions. Moreover, the additional low-ATP preincubation experiments further support our conclusion that Hrq1mon exhibits greater helicase activity than the high-molecular-mass Hrq1 fraction (Fig. S7). This assembly-dependent activation process may contribute to the lower apparent unwinding activity of Hrq1-oligo relative to that of Hrq1mon.

### ScRPA inhibits Hrq1 helicase activity in a concentration-dependent manner

Previous studies have shown that replication protein A (RPA) can stimulate the activities of some RecQ family helicases through interactions with ssDNA and/or the helicase (42, 43). However, ScRPA has been reported to inhibit Hrq1 unwinding (19, 25). As these conclusions were based on endpoint assays like EMSA, which cannot resolve the reaction dynamics in real time, this study employed stopped-flow FRET to monitor the unwinding process kinetically.

**Figure 6:**
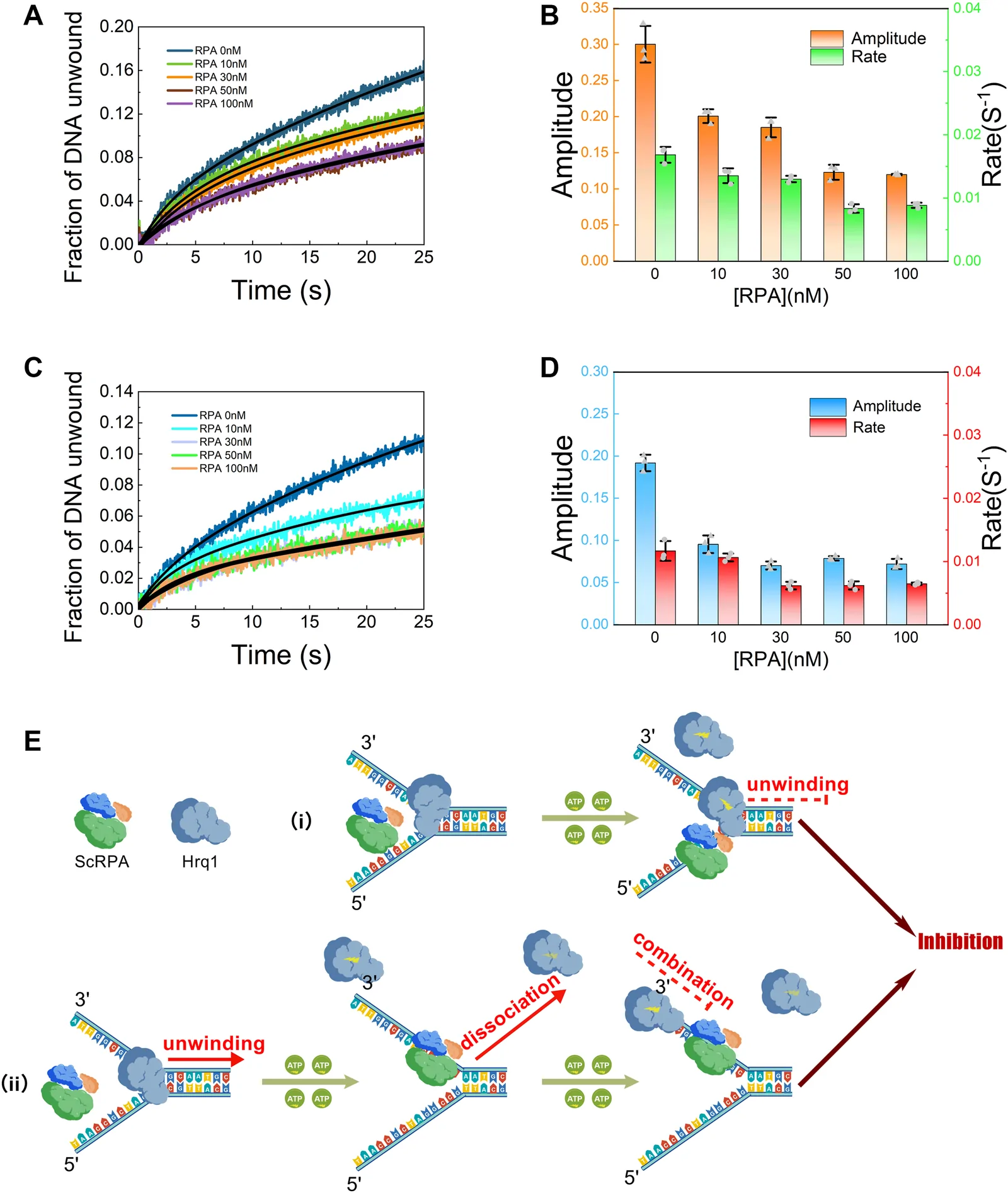
**ScRPA inhibits Hrq1 helicase activity.** Note: *A,* real time unwinding kinetics of the Hrq1 monomer in the presence of a ScRPA concentration gradient. Stopped-flow FRET was used to monitor the inhibitory effect of varying concentrations of ScRPA (0–100 nM) on the unwinding of the D20-3′dT25-Fk substrate (2 nM) by the Hrq1 monomer (100 nM). Reaction setup: Hrq1 and DNA were preincubated in channel 1 (Buffer A: 25 mM Tris-HCl, pH 7.5, 50 mM NaCl, 10 mM Mg^2+^, and 1 mM DTT), whereas 2 mM ATP and ScRPA were placed in channel two and rapidly mixed with the contents of channel one to initiate the reaction. The reaction was performed under multiple-turnover conditions, and the kinetic traces were fitted using a double-exponential function, as described in “Materials and Methods.” The curves reveal that ScRPA delays the unwinding process in a concentration-dependent manner. *B,* the unwinding amplitude and apparent total rate of the Hrq1 monomer decreased stepwise as the ScRPA concentration increased, reaching a plateau at ≥50 nM ([ScRPA]:[Hrq1] = 1:2). *C,* under the same experimental conditions, ScRPA inhibits the Hrq1-oligo more strongly than it inhibits Hrq1mon. This reaction was also performed under multiple-turnover conditions, and the unwinding kinetics was fitted using the double-exponential function. *D,* the total amplitude and rate of Hrq1-oligo reached a plateau at ≥30 nM ScRPA, suggesting that Hrq1-oligo is more sensitive to ScRPA inhibition under these conditions. *E,* dual-mechanism model for ScRPA inhibition of Hrq1 unwinding. Mechanistic diagram: (i) Steric hindrance: ScRPA binds to nascent 5′- ssDNA, blocking Hrq1 loading sites; (ii) Relay interruption: ScRPA occupies the 3′-ssDNA relay site, preventing subsequent Hrq1 binding. These two mechanisms synergistically inhibit the unwinding process. Created with BioGDP (61). Hrq1mon, monomeric Hrq1; ScRPA , ScRPA*, Saccharomyces. cerevisiae* replication protein A; Hrq1-oligo, higher-order oligomeric fraction.

Substrate D20-3′dT25-Fk (2 nM) was preincubated with 100 nM Hrq1mon or Hrq1-oligo at 37 °C for 5 min in the reaction buffer. The unwinding reaction was then initiated by rapid mixing with a solution containing 2 mM ATP and increasing concentrations of ScRPA. The results showed that ScRPA significantly inhibited the unwinding activity of both Hrq1mon and Hrq1-oligo (Fig. 6, *A* and *C*). However, the extent of inhibition differed between the two forms. For Hrq1mon, the inhibitory effect reached a plateau at ScRPA concentrations of 50 nM or higher, corresponding to an [ScRPA]:[Hrq1mon] ratio of 1:2 (Fig. 6*B*). By contrast, Hrq1-oligo appeared more sensitive to ScRPA-mediated inhibition, with the inhibitory effect reaching a plateau at a lower ScRPA concentration of 30 nM or higher (Fig. 6*D*).

Based on the two-syringe stopped-flow design,in which ScRPA and ATP were loaded into the second syringe, ScRPA could not access the 3′or 5′-ssDNA overhangs before mixing with the first syringe containing the preformed Hrq1-DNA complex. Furthermore, because Hrq1 (100 nM) was present at a 50-fold molar excess over the DNA substrate (2 nM), the 3′-ssDNA tail was likely occupied by Hrq1 at the start of the reaction (*t* = 0). Therefore, ScRPA inhibition likely began only after DNA unwinding had commenced, when duplex opening generated additional ssDNA regions. ScRPA could then bind newly exposed ssDNA or compete for the original overhang after dissociation of the initially bound Hrq1 molecule. We therefore speculate that ScRPA inhibits Hrq1-mediated unwinding through a combination of the following mechanisms (Fig. 6*E*).

(i) ScRPA is highly likely to bind to the substrate's 5′-ssDNA tail due to its high ssDNA-binding ability, thereby affecting the partially stable binding of the Hrq1–DNA complex or creating steric hindrance that interferes with the Hrq1 unwinding process.
(ii) Because Hrq1 shows weak processivity, multiple molecules are required to act sequentially on the 3′-ssDNA in a relay mechanism. ScRPA may rapidly occupy these critical sites, potentially preventing the binding of additional free Hrq1 molecules and blocking the multiple-protein “relay” unwinding process. The overall inhibitory effect of ScRPA is thus proposed to result from the combined effects of these mechanisms, targeting different stages of the unwinding process.

## Discussion

As key factors in maintaining genome stability, RecQ family helicases play essential roles in DNA replication, repair, and telomere homeostasis. In this study, we obtained highly purified Hrq1 using a baculovirus expression system in insect cells and systematically characterized its ATP dependent changes in assembly state and duplex DNA unwinding properties, thereby providing new experimental insights into the functional molecular mechanisms of yeast Hrq1 and its human homolog RECQL4.

First, we found that Hrq1 can stably exist in both monomeric and higher-order assembly states in a eukaryotic expression system, challenging the previous view that the assembly state of Hrq1 is determined primarily by the expression host. Earlier studies suggested that bacterially expressed Hrq1 predominantly forms heptamers (14), whereas eukaryotically expressed Hrq1 exists mainly as a monomer (15), and this difference has been attributed to differences in posttranslational modifications (15). However, the simultaneous isolation of both forms from the same insect cell expression system suggests that Hrq1 oligomerization may not be dictated solely by the expression system, but may instead reflect an intrinsic equilibrium among different assembly states of the protein.In addition, we found that ATP markedly shifts higher-order Hrq1 assemblies toward smaller species containing Hrq1 and that this redistribution is accompanied by increased helicase activity. These observations suggest that ATP may function not only as an energy substrate but also as an important signal regulating Hrq1 conformational or assembly state switching. This ATP dependent conformational “switch” resembles that reported for the BLM helicase (27, 44), but may differ from the behavior of WRN, which has been reported to function predominantly in oligomeric forms (45). We therefore speculate that higher-order Hrq1 assemblies could have noncatalytic or structural functions, such as acting as a molecular scaffold to recruit repair factors including Pso2 (20, 21), or contributing to the stabilization of structures associated with telomeres (46, 47). Consistent with observations for other RecQ family helicases, the dissociation of these higher-order assemblies induced by ATP may convert Hrq1 into monomer-like or lower-order assembly species that are catalytically competent for DNA unwinding. It also remains possible that a species of high molecular mass detected by SEC in the presence of long ssDNA represents a nucleoprotein complex formed by multiple Hrq1 molecules associated with a single DNA molecule, rather than a true preexisting oligomeric complex. Therefore, the functional relevance of higher-order Hrq1 assemblies may involve a balance between structural scaffolding and the generation of active monomeric units, rather than serving a strictly catalytic role. Such a regulatory strategy may provide an evolutionary advantage, allowing Hrq1 to respond flexibly to complex genomic contexts such as telomeric regions or interstrand crosslink lesions.

In addition, this study provides mechanistic insights into duplex DNA unwinding mediated by Hrq1. Consistent with this regulation by ATP, our analysis of ATPase activity under steady state conditions further supports functional coupling between DNA stimulated ATP hydrolysis and Hrq1 mediated DNA unwinding. Although the apparent ATPase parameters should be interpreted as steady state catalytic parameters rather than direct ATP binding constants, the decrease in K_m_,app for ATP in the presence of DNA and increase in *k*_cat_ indicate that forked DNA promotes a more catalytically active ATPase state (33, 48, 49). Importantly, the ATPase *k*_cat_ should not be directly equated with the DNA translocation rate at the single molecule level or the rate of base pair unwinding (40, 41, 50). Thus, the limited unwinding amplitude observed under conditions with a high enzyme-to-DNA ratio is more likely explained by low processivity, premature dissociation, or nonproductive binding, rather than by insufficient ATP hydrolysis alone (40, 51, 52). This interpretation is consistent with the enhanced unwinding observed under multiple turnover conditions (Fig. S4*B*) and further supports the proposed relay model for DNA unwinding (51, 52, 53). Because Hrq1 exhibits a strong dependence on forked DNA substrates, we initially hypothesized, based on other RecQ family helicases such as BLM, which uses a 5′-ssDNA tail to drive processive unwinding through strand switching (27, 54), that Hrq1 similarly requires a 5′-ssDNA tail for unwinding mediated by strand switching. However, single-molecule fluorescence resonance energy transfer analysis revealed no fluorescence oscillations characteristic of strand switching (55). Instead, Hrq1 was observed to unwind the DNA duplex without detectable strand switching behavior (Fig. S6, *A*–*C*). These findings suggest that Hrq1 likely uses the 5′-ssDNA tail primarily to stabilize binding at the ssDNA/ds DNA junction, thereby enhancing enzyme loading efficiency or prolonging residence time. Kinetic analyses under single turnover conditions further showed that Hrq1 preloaded onto a 3′-ssDNA tail was unable to independently sustain unwinding of long duplex DNA substrates. Together with the overall weak processivity of Hrq1, these observations led us to propose that Hrq1 may operate through a “relay” unwinding mechanism. In this model, Hrq1 functions as a monomeric catalytic unit that locally disrupts base pairing through direct unwinding. Because of its limited intrinsic processivity, a single Hrq1 molecule is insufficient to continuously unwind long duplex DNA regions. Instead, efficient unwinding requires a sufficiently long 3′-ssDNA tail (≥45 nt) that provides additional loading sites, allowing multiple Hrq1 molecules to bind sequentially and replace one another during unwinding, or permitting free Hrq1 molecules to rebind newly exposed 3′ tail regions after dissociation of preceding molecules. In this way, robust overall unwinding can be achieved. The inhibitory effect of ScRPA on Hrq1 further supports this model, as competition for ssDNA binding sites would be expected to interfere with Hrq1 loading and sequential handoff, suggesting that efficient Hrq1 unwinding depends on the dynamic cooperation of multiple molecules. From a biological perspective, the relay model is highly consistent with the potential physiological functions of Hrq1. For example, during late S phase in yeast telomeric regions, the 3′ overhang can extend to 50 to 100 nt (17, 56), which may provide a natural platform for sequential Hrq1 loading, thereby facilitating the removal of accumulated G4 structures and preventing abnormal telomere shortening (57, 58, 59). Likewise, during interstrand crosslink repair, long ssDNA gaps generated during lesion processing (>100 nt) may provide a similar platform for Hrq1 to remove D-loops, G4 structures, or other secondary DNA structures through repeated relay-like loading events (60).

This study still has several limitations. The precise stoichiometry and structures at atomic resolution of the higher-order Hrq1 assemblies remain unresolved, and their assembly interfaces and transitions in conformation and assembly state induced by ATP require further investigation. In addition, the proposed “relay” unwinding model is based primarily on biochemical and single molecule kinetic analyses and has not yet been directly validated by *in vivo* functional studies or imaging in real time. Future studies integrating cryoelectron microscopy, fluorescence imaging at the single molecule level, and genetic analyses should provide a more comprehensive understanding of the dynamic mechanisms and physiological functions of Hrq1 and its human homolog, RECQL4.

Taken together, our study reveals changes in the apparent assembly state of Hrq1 induced by ATP supports a model in which Hrq1 unwinds DNA through a relay mechanism characterized by low processivity and involving the successive action of multiple Hrq1 molecules. These findings expand our understanding of the functional regulation of RecQ family helicases and provide a conceptual framework for investigating the molecular mechanisms underlying genetic disorders associated with RECQL4 and their potential therapeutic implications.

## Experimental procedures

### Reagents and buffers

All chemicals used in this study were of analytical grade and, unless otherwise specified, were purchased from Sigma-Aldrich. Buffers were prepared using ultrapure water produced with an ELGA PURELAB Classic system (resistivity > 18.2 MΩ cm) and were filtered through 0.45-μm membrane filters to remove particulate matter.

### Oligonucleotide substrates

Oligonucleotides used in stopped-flow FRET experiments were purchased from General Biological Co., Ltd, and their sequences are provided in Table S1. The double-stranded DNA substrates were constructed by annealing two complementary oligonucleotides: a BHQ1-labeled strand synthesized in the 5′→3′ direction and a complementary FAM-labeled strand. Black Hole Quencher 1 (BHQ1) denotes Black Hole Quencher 1, and FAM denotes fluorescein amidite. The molar extinction coefficients of the oligonucleotides were calculated using the nearest-neighbor method, and the concentrations of the single-stranded oligonucleotides were determined spectrophotometrically.

The BHQ1-labeled strand was mixed in excess with the FAM-labeled strand at a molar ratio of 1.2:1. The mixture was incubated in annealing buffer (25 mM Tris-HCl, pH 7.5, and 10 mM MgCl_2_) at 95 °C for 5 min and then slowly cooled to room temperature over approximately 3 h. The excess BHQ1-labeled strand ensured complete annealing of the FAM-labeled strand, thereby minimizing background fluorescence from free FAM-labeled ssDNA.

The protein trap oligonucleotide used in the single-turnover unwinding kinetic experiments was a 56-nt poly(dT) oligonucleotide (dT56). All assembled oligonucleotide substrates were stored at −20 °C.

### Production of recombinant *S. cerevisiae* Hrq1 and its catalytically inactive mutant Hrq1-K318A using the baculovirus expression system

The gene sequence encoding Hrq1 helicase was cloned into the pFastBac Dual vector downstream of the polyhedrin promoter using the SpeI restriction site, with a 10×His affinity tag fused to the N terminus of Hrq1. This resulted in the recombinant plasmid pFastBac Dual-10×His-Hrq1. Additionally, the superfolder green fluorescent protein (sfGFP) reporter gene was inserted downstream of the P10 promoter in the same vector, generating the dual-expression recombinant vector pFastBac Dual-sfGFP-10×His-Hrq1.

For the catalytically inactive mutant, site-directed mutagenesis was performed catalytically inactive mutant, site-directed mutagenesis was performed using pFastBac Dual-sfGFP-10×His-Hrq1 as the template by inverse PCR with the primers Hrq1-K318A-F (5′-AGCAGCGGCGCGAG-CCTGATTTATCAGCTG) and Hrq1-K318A-R (5′-AATCAGGCTCGCGCCGCTGCTGGTGCTGGT). After digestion with DpnI to remove the parental template plasmid, the mutant plasmid pFastBac Dual-sfGFP-10×His-Hrq1-K318A was obtained.

The recombinant vector was transformed into DH10Bac chemically competent cells (Weidi Bio) and plated onto LB agar plates containing X-Gal/IPTG for blue-white screening (50 μg/ml kanamycin, 7 μg/ml gentamicin, and 10 μg/ml tetracycline), The plates were incubated at 37 °C for 48 h. White colonies were selected, cultured, and recombinant bacmid DNA was isolated according to the instructions provided with the Bac-to-Bac Baculovirus Expression System (Thermo Fisher Scientific). The recombinant bacmid was verified by PCR using the universal primers pFastBac-F and pFastBac-R (5′-GTTTTCCCAGTCACGAC-3′ and 5′-CAGGAAACAGCTATGAC-3′, respectively) at an annealing temperature of 58 °C.

*Spodoptera frugiperda* (Sf9) insect cells were cultured to the logarithmic growth phase (viability > 95%) and adjusted to a density of 1.2 × 10^6^ cells/ml. A 3 ml aliquot of the cell suspension was mixed with 8 μg of recombinant bacmid DNA and 8 μl of Cellfectin II (Merck), and the cells were transfected in serum-free medium (ESF 921, Expression Systems) at 28 °C with shaking at 130 rpm for 96 h. The P0 viral supernatant was harvested and used to infect Sf9 cells at an MOI of 0.1. The infected cells were incubated at 28 °C for 72 h to generate the P1 viral stock. The P1 virus was then used to infect Sf9 cells at an MOI of 0.5 to obtain a high-titer P2 virus stock.

The P2 virus was used to infect High Five (Hi5) cells at an infection ratio of 1:80 for large-scale protein expression. Cell density, viability (determined by trypan blue exclusion; viability >90%), fluorescence intensity, and cell morphology (assessed *via* microscopy) were monitored daily. The cells were harvested by centrifugation at 1000×*g* for 10 min, flash-frozen in liquid nitrogen, and stored at −80 °C until protein purification.

### Production and expression of recombinant ScRPA using the baculovirus expression system

To express the *S. cerevisiae* replication protein A (ScRPA) heterotrimer in a eukaryotic system, a baculovirus expression system was used. Because ScRPA consists of three subunits—RPA70, RPA32, and RPA14—their coexpression and assembly had to be coordinated. First, the RPA14 subunit was cloned downstream of the P10 promoter in the pFastBac Dual vector using the NcoI restriction site,and the C-terminally Strep-tagged RPA70 subunit was cloned downstream of the polyhedrin promoter using the SpeI restriction site, resulting in pFastBac Dual-RPA14-RPA70-Strep-TagⅡ. The RPA32 subunit was then inserted downstream of the polyhedrin promoter using the SpeI restriction site to generate the pFastBac Dual-sfGFP-RPA32 vector.

After the recombinant plasmids pFastBac Dual-RPA14-RPA70-Strep-tag II and pFastBac Dual-sfGFP-RPA32 were successfully constructed and verified, P2 viral stocks were prepared as described in “Materials and Methods 4.3”. For coexpression of the RPA complex, the two recombinant viruses were used to infect Hi5 cells at specific MOIs (RPA14-RPA70, MOI 1:80; RPA32, MOI 1:100). The infected cells were cultured at 28 °C with shaking at 130 rpm for 72 h. Following protein expression, the cells were harvested and frozen as described in “Materials and Methods 4.3” for subsequent protein purification.

### Purification of the ScRPA complex

ScRPA purification followed the Hrq1 workflow with modifications to the affinity resin, buffer pH, and elution strategy. Approximately 10 g of cell pellet (from 500 ml of Hi5 cells) was thawed and resuspended in 70 ml of ice-cold Strep Lysis Buffer (25 mM Tris-HCl, pH 7.9, 400 mM NaCl, and 10% glycerol) containing the same protease inhibitors. After lysis and clarification as above, the supernatant was incubated with 5 ml of pre-equilibrated Strep-Tactin for 3 h at 4 °C. The resin was washed by centrifugation, transferred to a 20-mL gravity-flow column, and sequentially washed with: 5 CV of Strep Lysis Buffer, 5 CV high-salt buffer (25 mM Tris-HCl,pH 7.9, 1 M NaCl, and 10% glycerol) to remove nucleic acids, and 5 CV of Strep Lysis Buffer.

The protein was specifically eluted with 20 ml of Strep Lysis Buffer containing 5 mM D-biotin. Fractions verified by SDS-PAGE to contain ScRPA were pooled and concentrated to approximately 3 ml, then exchanged into low salt buffer (25 mM Tris-HCl,pH 7.9, 100 mM NaCl, and 10% glycerol). Anion exchange chromatography (HiTrap Q FF, Cytiva; flow rate 1 ml/min; linear NaCl gradient using an ÄKTA system) and size-exclusion chromatography (Superdex 200 Increase 10/300 Gl column, Cytiva; 25 mM Tris-HCl, pH 7.9, 400 mM NaCl, and 10% glycerol; flow rate 0.3 ml/min) were performed as for Hrq1. ScRPA-containing fractions were identified by SDS-PAGE and absorbance at 280 nm, pooled, and concentrated. Final protein purity exceeded 95% (Fig. S1). ScRPA samples were stored in gel filtration buffer containing 50% glycerol and 0.5 mM DTT, flash-frozen in liquid nitrogen, and stored at −80 °C.

### Purification of Hrq1 helicase and catalytically inactive mutant Hrq1-K318A

Approximately 19 g of cell pellet (derived from 1 L of Hi5 insect cell culture) was thawed on ice. The thawed cells were resuspended in 100 ml of ice-cold Ni Lysis Buffer (50 mM Na-Hepes, pH 7.6, 400 mM NaCl, 10 mM imidazole, and 10% glycerol), supplemented with a protease inhibitor cocktail at final concentrations of 1 μg/ml aprotinin, 2 μg/ml leupeptin, 0.5 μg/ml pepstatin, and 0.4 mM PMSF. Cell lysis was performed using high-pressure homogenization (1000 bar, three passes), followed by intermittent sonication (150 W, 3 s on and 3 s off for 5 min).

The lysate was clarified by centrifugation at 10,000 rpm for 60 min at 4 °C, and the supernatant was collected. The supernatant was incubated with 5 ml of Ni-NTA resin (Tiandirenhe), equilibrated with Ni Lysis Buffer, for 3 h at 4 °C with gentle agitation. After incubation, the resin was washed multiple times by centrifugation (1000×*g* for 5 min) until clear. The resin, now bound to the target protein, was transferred to a 20-mL gravity-flow chromatography column.

The column was sequentially washed with five column volumes of Ni Lysis Buffer to remove nonspecifically bound proteins, five column volumes of high salt buffer (50 mM Na-Hepes, pH 7.6, 1 M NaCl, and 10% glycerol) to eliminate nucleic acids, and five column volumes of Ni Lysis Buffer for re-equilibration. The target protein was eluted using a stepwise imidazole gradient (50–500 mM; 10 ml per step) in Ni Lysis Buffer. The Eluted fractions were analyzed by SDS-PAGE, and those containing the target protein were pooled and concentrated to approximately 5 ml.

Buffer exchange was performed using low salt buffer (50 mM Na-Hepes, pH 7.6, 100 mM NaCl, and 10% glycerol). The sample was loaded at a flow rate of 1 ml/min onto a HiTrap Q FF anion exchange column (Cytiva), equilibrated with the same low-salt buffer. Protein elution was performed using a linear NaCl gradient on an ÄKTA chromatography system. Fractions were analyzed by SDS-PAGE, and those corresponding to the target protein peak were pooled and concentrated to approximately 500 μl. The concentrated sample was subsequently loaded onto a Superose 6 Increase 10/300 Gl column (Cytiva), equilibrated with gel filtration buffer (50 mM Na-Hepes, pH 7.6, 400 mM NaCl, and 10% glycerol), and eluted at a flow rate of 0.3 ml/min,with 0.3 ml collected per fraction. Based on the size-exclusion chromatography elution profile, together with SDS-PAGE analysis and absorbance at 280 nm, the monomeric and multimeric forms of Hrq1 were identified, collected separately, and concentrated. The final protein samples were stored in gel filtration buffer supplemented with 50% glycerol and 0.5 mM DTT, snap-frozen in liquid nitrogen, and stored at −80 °C for long-term storage. The catalytically inactive mutant Hrq1-K318A was purified using an identical procedure. Final protein purity exceeded 90% (Fig. S3*A*).

### SEC

SEC was performed at 25 °C using a Cytiva ÄKTA FPLC system equipped with a Superose 6 Increase 10/300 Gl analytical column The column void volume was determined using Blue Dextran 2000 and was measured was 7.42 ml. The column was equilibrated with buffer containing 25 mM Tris-HCl (pH 7.5), 100 mM NaCl, 10 mM MgCl_2_, (Fig. 1, *A* and *B*:−DTT; Fig. 1*C*:+DTT), and 10% (v/v) glycerol. A total of 120 μl of Hrq1 protein was loaded onto the column and eluted isocratically at a flow rate of 0.3 ml/min, while UV absorbance was continuously monitored at 280 nm.

For molecular mass calibration, a Bio-Rad gel filtration protein standard set was used, consisting of thyroglobulin (670 kDa), bovine γ-globulin (158 kDa), chicken ovalbumin (44 kDa), horse myoglobin (17 kDa), and vitamin B_12_ (1.35 kDa). For each protein standard and Hrq1 sample, the partition coefficient, Kav was calculated according to the equation:

$$\text{Kav}=\left(\text{Ve}-V_{0}\right)/\left(\text{Vt}-V_{0}\right)$$

where Ve is the elution volume of the protein peak, void volume is the void volume of the column, and Vt is the total column volume. A standard calibration curve was then generated by plotting the logarithm of molecular weight, log_10_R, against the corresponding Kav values of the protein standards, followed by linear regression to obtain the equation:

$$\log_{10}\;R=\text{aKav}+b$$

where R represents the apparent molecular mass of the protein. The apparent molecular mass of Hrq1 was then calculated by substituting its experimentally determined Kav value into the fitted equation. To ensure reproducibility and accuracy, the column void volume was routinely verified prior to SEC analysis.

### DLS

DLS measurements were carried out using a NanoPartica SZ-100 instrument (HORIBA Scientific). All measurements were performed at 25 °C, with scattered light collected at a detection angle of 90°. Buffer A contained 25 mM Tris-HCl, pH 7.5, 100 mM NaCl, 1 mM DTT, and 10 mM MgCl_2_, and was filtered through a 0.1-μm membrane filter before use. Protein samples contained 1 μM Hrq1mon or Hrq1-oligo.

Before measurement, the protein samples were incubated with 5 mM ATP and 3 μM poly(dT) oligonucleotides of defined lengths (10 nt or 56 nt) at room temperature for 10 min to allow complex formation. During data acquisition, the instrument automatically recorded the scattered-light autocorrelation function, with one data point collected every 10 s. raw data were analyzed using the accompanying Dynamics v7.0 software (HORIBA Scientific; https://www.horiba.com/). The Rh was determined using either the cumulant method or distribution fitting models, and the apparent molecular mass was subsequently estimated according to the empirical equation:

$$\text{Mw}\left(\text{kDa}\right)={\left(1.68\times \text{Rh}\right)}^{2.34}$$

### ATPase activity assay

ATPase activity assays were performed to evaluate the ATP-hydrolyzing capability of the protein, as previously described (28). Briefly, reactions containing 100 nM helicase were carried out in the presence of 1 μM forked DNA substrate (D20-3′dT25-Fk; sequence listed in Table S1) and 2 mM ATP. Measurements were conducted using a CLARIOstar Plus multifunctional microplate reader (BMG LABTECH). All reactions were performed in Buffer A at 25 °C in a total volume of 50 μl and in triplicate. The kinetic parameters *K*_m_ and *k*_cat_ were determined by fitting the experimental data to the Michaelis–Menten equation using Origin 9.0 software (OriginLab Corporation; https://www.originlab.com/).

### Stopped-flow FRET measurements

Stopped-flow (stopped-flow FRET) measurements were used to characterize the kinetics of Hrq1-mediated DNA unwinding. A dual-labeled DNA substrate was used, carrying 6-carboxyfluorescein (FAM) at the 5′ end as the donor fluorophore and BHQ1 at the 3′ end as the acceptor quencher.

Experiments were performed using a Bio-Logic SFM-400 stopped-flow mixing system equipped with an FC-15 observation cell (1.5-mm path length; Bio-Logic), coupled to a MOS-450/AF-CD optical detection module and a 150-W mercury-xenon light source. Excitation was set at 492 nm with a 2-nm slit width, and fluorescence emission was collected through a D525/20 high-pass filter (Chroma Technology Co.; center wavelength, 525 nm; bandwidth, 20 nm). The temporal resolution for data acquisition was 0.5 ms.

A two-syringe configuration was used. Hrq1 helicase (100 nM) and the dual-labeled DNA substrate (2 nM) were preincubated in the unwinding reaction Buffer A at 37 °C for 5 min in syringe 1. The reaction was initiated by rapid mixing with syringe two containing 2 mM ATP in the absence or presence of the competitive inhibitor dT_56_ (1 μM). Unless otherwise indicated, the final concentrations after mixing were 100 nM Hrq1, 2 nM DNA substrate, 2 mM ATP, and 1 μM dT56. All buffers and nucleotide-containing solutions were filtered through 0.45-μm membrane filters and degassed by ultrasonication (40 kHz for 15 min) before use.

To establish a quantitative relationship between the fluorescence signal and the apparent fraction of DNA unwound, the initial fluorescence signal of the fully annealed dual-labeled double-stranded DNA substrate was defined as the 0% unwound baseline (F_min). The fluorescence signal of the corresponding FAM-labeled single-stranded DNA at the same concentration was measured under the same instrumental conditions and defined as the signal corresponding to 100% unwound DNA (F_max). The fraction of DNA unwound at each time point was calculated as:

Fractionunwound=(F(t)−F_min)/(F_max-F_min)

where F(t) is the fluorescence intensity at a given time point, F_min is the fluorescence intensity of the fully annealed double-stranded substrate, and F_max is the fluorescence intensity corresponding to the fully unwound single-stranded product state. Thus, the Y-axis value represents the fraction of DNA unwound and ranges from 0 to 1 and represented the ensemble-averaged apparent fraction of DNA unwound, rather than the absolute base-pair unwinding rate or single-molecule translocation rate. Each kinetic trace was repeated at least eight times and averaged to reduce noise. Data were fitted by nonlinear least-squares analysis using OriginPro 2024b (OriginLab) according to the following biexponential equation:

$$A\left(t\right)=A_{0}+A_{1}\;\exp\left[-\left(t-t_{0}\right)/t_{1}\right]+A_{2}\;\exp\left[-\left(t-t_{0}\right)/t_{2}\right]$$

where A(t) is the fraction of unwound dsDNA as a function of time; A_0_ is the baseline; A1 and A2 are the amplitudes of the fast and slow phases, respectively (A_1_ + A_2_ = total unwinding amplitude); and t1 and t2 are the characteristic time constants (s) for the two phases. The fast and slow phase rate constants were calculated as k_fast_ = A1/t1 and k_slow_ = A_2_/t_2_, respectively, and the apparent overall rate was defined as k_obs_ = k_fast_ + k_slow_. It should be noted that kobs represents an ensemble-level apparent unwinding rate reflecting the overall fraction of DNA unwound per unit time under the experimental conditions, including contributions from base-pair separation, enzyme translocation along ssDNA, enzyme binding/dissociation, and possible rebinding events in multiple-turnover conditions. It should not be interpreted as the absolute single-molecule DNA translocation rate or base-pair unwinding velocity.

### Single-molecule fluorescence resonance energy transfer assays

Single-molecule fluorescence resonance energy transfer measurements were performed using a custom-built total internal reflection fluorescence microscope. Coverslips and microscope slides were thoroughly cleaned using a mixture of sulfuric acid, hydrogen peroxide, acetone, and sodium ethoxide, followed by surface passivation of the coverslips with 99% methoxy-polyethylene glycol (mPEG-5000; Laysan Bio, Inc.) and 1% biotin-PEG (biotin-PEG-5000; Laysan Bio, Inc.).

A microfluidic flow chamber was assembled using a microscope slide and a PEG-coated coverslip. Streptavidin (10 mg/ml in Buffer A) was introduced into the chamber and incubated for 10 min to allow binding to the biotinylated surface. After washing, 50 pM S17-3′dT25-FK DNA substrate was introduced and incubated for 10 min to allow immobilization. Unbound DNA molecules were removed by washing with imaging buffer containing 30 mM Tris-HCl, pH 7.5, 90 mM NaCl, 1 mM DTT, and 10 mM MgCl_2_, 0.8% D-glucose, 1 mg/ml glucose oxidase, 0.4 mg/ml catalase, and 4 mM Trolox. Image acquisition was initiated before the introduction of monomeric Hrq1 and ATP into the chamber. Single-molecule fluorescence measurements were performed at a constant temperature of 25 °Cwith an exposure time of 100 ms.

For analysis of single-molecule unwinding events, DNA molecules displaying fluorescence signals from both the donor and acceptor fluorophores (Cy3 and Cy5) were first identified. Fluorescence trajectories were processed using the instrument-associated analysis software. The dynamic unwinding behavior of Hrq1 helicase at the single-molecule level was characterized by monitoring changes in the donor and acceptor fluorescence intensities and the corresponding FRET efficiency over time. Photobleaching behavior was used to assist in fluorophore validation and trajectory selection.

## Data availability statement

The data that support the findings of this study are available from the corresponding author upon reasonable request.

## Supporting information

This article contains supporting information.

## Conflict of interest

The authors declare that they have no conflicts of interest with the contents of this article.
