# Supplementary material for: Mechanistic study of ATP-dependent regulation of the Hrq1 oligomeric state and its DNA unwinding activity in Saccharomyces cerevisiae
Source: J Biol Chem. 2026 Aug 7;302(9):113404. doi: 10.1016/j.jbc.2026.113404 (PMC13571135; doi:10.1016/j.jbc.2026.113404)
Supplement: Supplementary file 1 [file mmc1.docx]

**Figure S1**

**Figure S1. SDS-PAGE Analysis of Recombinant *Saccharomyces cerevisiae* RPA (ScRPA) Protein Purified by Three-Step Chromatography.**

Note: The three clearly visible major bands correspond to the subunits of the heterotrimeric complex: RPA70 (approximately 70 kDa), RPA34 (approximately 34 kDa), and RPA14 (approximately 14 kDa), confirming the successful purification of the intact complex.

**Figure S2**

**Figure S2. **Hydrodynamic radius of Hrq1 in the absence or presence of ATP and ssDNA, as determined by analysis of data from dynamic light scattering measurements performed at room temperature** (**see** Materials and Methods).**

**Figure S3**

**Figure S3. General analysis of Hrq1 helicase activity.**

Note: Unless otherwise indicated, the reactions shown in panels B–H were performed in the absence of a protein trap and therefore under multiple-turnover or steady-state conditions. For panels showing stopped-flow DNA unwinding traces, the curves were fitted using the biexponential equation described in the “Materials and Methods”. Panel E was used to evaluate fluorescence quenching by metal ions and was not subjected to kinetic fitting. **(A)** SDS-PAGE analysis of wild-type Hrq1 and its catalytically inactive mutant, showing protein purity of greater than 95%. Mon-KA and oligo-KA denote the monomeric and oligomeric forms of the Hrq1-K318A mutant, respectively; mon and oligo denote the monomeric and oligomeric forms of wild-type Hrq1, respectively.**(B)** Temperature dependence of Hrq1 helicase activity. Reactions were carried out over a temperature range of 25–42 °C under fixed conditions of 50 mM NaCl, 10 mM MgCl_2_, 1 mM DTT, 25 mM Tris-HCl (pH 7.6), and 1 mM ATP. **(C)** pH dependence of Hrq1 helicase activity. Assays were performed in 25 mM Tris-HCl buffer (pH 6.5–8.5) containing 50 mM NaCl, 10 mM MgCl_2_, and 1 mM DTT at a constant temperature of 37 ℃. **(D)** Effect of increasing DTT concentration on Hrq1 helicase activity. Reactions were conducted in 25 mM Tris-HCl (pH 7.6), 50 mM NaCl, 10 mM MgCl_2_, and 1 mM ATP, with DTT concentrations varied as indicated. **(E)** Fluorescence quenching induced by Zn²⁺ and Ni²⁺ under in multiple-turnover conditions. **(F)** Effect of Mg^2+^ concentration on the kinetic curves of ScPif1. **(G)** Effect of ATP concentration on ScPif1 helicase activity under multiple-turnover conditions. **(H)** DNA-unwinding kinetics of Hrq1 under **steady-state, multiple-turnover conditions**.

**Figure S4**

**Figure S4. Dependence of Hrq1 Helicase Activity on the Length of the 5' Single-Stranded DNA (ssDNA) Overhang.**

Note: **(A)** Under single-turnover conditions in the presence of the protein capture oligonucleotide dT56, Hrq1 helicase activity was significantly inhibited. The maximum unwinding amplitude for fork/overhang substrates with different 5'-ssDNA tail lengths (0, 1, 5, or 25 nt) did not exceed 2.5%. This indicates that prebound Hrq1 is unable to independently unwind the substrate. Although the kinetic curves show a length-dependent trend for the 5'-ssDNA tail, the low signal-to-noise ratio makes reliable quantitative analysis difficult. Therefore, additional multiple-turnover kinetic experiments are required to further validate Hrq1’s dependence on forked structures. **(B)** Comparison of single-turnover and multiple-turnover unwinding kinetics. In the presence of dT56, free or dissociated Hrq1 was trapped, resulting in minimal unwinding. In contrast, under multiple-turnover conditions without dT56, Hrq1 molecules were able to rebind to the DNA substrate or newly exposed 3'-ssDNA regions, leading to a marked increase in the fraction of DNA unwound. **(C)** Under multiple-turnover conditions, when Hrq1 unwound forked substrates with a **25-nt random-sequence 5′ tail** (D20-**5′Random25-Fk**)or a **25-nt homopolymeric dT 5′ tail** (D20-**3′dT25-Fk**),no significant difference in unwinding amplitude (Random: 29.14 ± 1.19% versus dT: 30.03 ± 2.53%) or **in the unwinding rate (<15% difference)**was observed. This suggests that Hrq1’s helicase activity is not significantly affected by the sequence composition of the 5'-ssDNA tail, ruling out sequence preference as a factor. All stopped-flow unwinding traces were fitted using the biexponential equation described in “Materials and Methods”.

**Figure S5**

**Figure S5.** **Dependence of Hrq1 Helicase Efficiency on 3'-ssDNA Tail Length under Single-Turnover Conditions—Supporting the "Hand-off Cooperative" Mechanism.**

Note: In single-turnover unwinding kinetics, when the 3'-ssDNA tail length of the substrate was ≥45 nt (theoretically capable of binding at least two Hrq1 monomers), the unwinding amplitude of Hrq1 was significantly higher **than that observed for** forked substrates with 3'-tails of only 15 nt or 25 nt. This result suggests that a 20-bp double-stranded DNA region requires two or more Hrq1 monomers to cooperate **to achieve efficient unwinding**.These data directly confirm the central role of the "hand-off cooperative" mechanism in Hrq1 helicase activity.

**Figure S6**

**Figure S6. Single-molecule FRET assays validate the direct unwinding mode of Hrq1.**

Note: **(A)** Schematic illustration of the 17-bp forked substrate labeled with Cy3 and Cy5 fluorophores at the junction; **(B)** Representative images showing the disappearance of Cy5 spots; **(C)** Representative single-molecule fluorescence trajectories. The Cy5 signal drops directly to zero, followed by a transient increase in and subsequent loss of the Cy3 **signal**, **with no strand-switching oscillations observed**, indicating a direct unwinding mode.

**Figure S7**

**Figure S7**. Effect of ATP pre-incubation on Hrq1 helicase activity under low ATP conditions and multiple-turnover conditions.

DNA unwinding was measured using the D20S25 substrate. Hrq1 was pre-incubated with 5 µM or 10 µM ATP prior to reaction initiation, and helicase activity was monitored as the fraction of DNA unwound over time. ATP pre-incubation enhances Hrq1 unwinding activity in a concentration-dependent manner compared to non-pre-incubated controls. Under multiple-turnover conditions, ATP-dependent activation of Hrq1 was further examined at micromolar ATP concentrations. Pre-incubation with 5 µM or 10 µM ATP increased DNA unwinding amplitude by 58.05 ±4.27% and 28.64±1.91%, respectively. These results demonstrate that ATP-mediated activation of Hrq1 occurs even at micromolar ATP concentrations, and is likely driven by structural reorganization rather than the catalytic effect of millimolar ATP during the reaction. Non-pre-incubation controls: (Hrq1-oligo)+D20S25, 5 µM ATP; (Hrq1-oligo)+D20S25, 10 µM ATP. ATP pre-incubation conditions: (Hrq1-oligo)+5 µM ATP, D20S25; (Hrq1-oligo)+10 µM ATP, D20S25.
