# Supplementary material for: Mechanistic study of ATP-dependent regulation of the Hrq1 oligomeric state and its DNA unwinding activity in Saccharomyces cerevisiae
Source: J Biol Chem. 2026 Aug 7;302(9):113404. doi: 10.1016/j.jbc.2026.113404 (PMC13571135; doi:10.1016/j.jbc.2026.113404)
Supplement: Supplementary file 2 [file mmc2.docx]

**Table S1. Name, sequence and schematic diagram of the oligonucleotide substrate used in the study.**
